# Widespread dissolved inorganic carbon-modifying toolkits in genomes of autotrophic *Bacteria* and *Archaea* and how they are likely to bridge supply from the environment to demand by autotrophic pathways

**DOI:** 10.1128/aem.01557-23

**Published:** 2024-02-01

**Authors:** Kathleen M. Scott, Ren R. Payne, Arin Gahramanova

**Affiliations:** 1 Integrative Biology Department, University of South Florida, Tampa, Florida, USA; Washington University in St. Louis, St. Louis, Missouri, USA

**Keywords:** autotroph, carbon fixation, carbonic anhydrase, carbon dioxide concentrating mechanism

## Abstract

Using dissolved inorganic carbon (DIC) as a major carbon source, as autotrophs do, is complicated by the bedeviling nature of this substance. Autotrophs using the Calvin-Benson-Bassham cycle (CBB) are known to make use of a toolkit comprised of DIC transporters and carbonic anhydrase enzymes (CA) to facilitate DIC fixation. This minireview provides a brief overview of the current understanding of how toolkit function facilitates DIC fixation in *Cyanobacteria* and some *Proteobacteria* using the CBB and continues with a survey of the DIC toolkit gene presence in organisms using different versions of the CBB and other autotrophic pathways (reductive citric acid cycle, Wood-Ljungdahl pathway, hydroxypropionate bicycle, hydroxypropionate-hydroxybutyrate cycle, and dicarboxylate-hydroxybutyrate cycle). The potential function of toolkit gene products in these organisms is discussed in terms of CO_2_ and HCO_3_^−^ supply from the environment and demand by the autotrophic pathway. The presence of DIC toolkit genes in autotrophic organisms beyond those using the CBB suggests the relevance of DIC metabolism to these organisms and provides a basis for better engineering of these organisms for industrial and agricultural purposes.

## INTRODUCTION

The first step of the biological carbon cycle is the fixation of dissolved inorganic carbon (DIC; CO_2_ + HCO_3_^−^ + CO_3_^=^) by organisms consuming it via autotrophic and anaplerotic pathways [reviewed in reference (1)]. The entry of DIC into the biological carbon cycle is complicated by aspects of DIC that make it a tricky growth substrate. The composition of DIC is sensitive to pH; CO_2_ dominates at low pH, HCO_3_^−^ at circumneutral pH, and CO_3_^=^ at alkaline pH. The different forms of DIC have profound differences in geometry and charge (linear neutral CO_2_ vs. trigonal planar anions HCO_3_^−^ and CO_3_^=^). Due to these differences in geometry and charge, enzymes are specific to different forms of DIC (Table 1). Many key autotrophic enzymes are specific to CO_2_, which is problematic since HCO_3_^−^ is the most abundant form at physiological, circumneutral pH (2). Using HCO_3_^−^ has its own complications; CO_2_ diffuses through cell membranes more rapidly than HCO_3_^−^ (3), due to higher permeability in phospholipid bilayers (4) and aquaporins (5). These difficulties in using CO_2_ or HCO_3_^−^ are exacerbated by the slow rate of uncatalyzed interconversion between them, relative to metabolism (2). Nature has responded to these challenges with a toolkit consisting of several carbonic anhydrase enzymes [EC 4.2.1.1; (6)] and DIC transporters (7, 8).

**TABLE 1:** Substrate specificities of DIC-metabolizing enzymes from autotrophic and anaplerotic pathways

Enzyme	EC	Substrate	References
Malic enzyme^*a*^	1.1.1.38 1.1.1.39 1.1.1.40	CO_2_	(9, 10)
Isocitrate dehydrogenase	1.1.1.41	CO_2_	(11)
Isocitrate dehydrogenase with carboxylating factor for IDH	6.4.1.7 and 1.1.1.41	HCO_3_^-^	(12)
Pyruvate synthase	1.2.7.1	CO_2_	(13)
2-Oxoglutarate synthase	1.2.7.3	CO_2_	(13)
Carbon monoxide dehydrogenase/ acetyl-CoA synthase	1.2.7.4/2.3.1.169	CO_2_	(14)
Formylmethanofuran dehydrogenase	1.2.7.12	CO_2_	(15)
Formate dehydrogenase	1.17.1.10	CO_2_	(16)
Phosphoenolpyruvate carboxylase	4.1.1.31	HCO_3_^-^	(17–19)
Phosphoenolpyruvate carboxykinase^*a*^	4.1.1.32 4.1.1.38 4.1.1.49	CO_2_	(20)
Ribulose 1,5-bisphosphate carboxylase/oxygenase	4.1.1.39	CO_2_	(21)
Pyruvate carboxylase	6.4.1.1	HCO_3_^-^	(20)
Acetyl-CoA/propionyl-CoA carboxylase	6.4.1.2/6.4.1.3	HCO_3_^-^	(22)
Oxaloacetate decarboxylase (Na^+^ extruding)^*a*^	7.2.4.2	HCO_3_^-*b*^	

^*a*^ Though these enzymes generally operate under physiological conditions as decarboxylases (23), they have been shown to be capable of acting as carboxylases (24, 25).

^*b*^ The DIC substrate for this enzyme have not been directly measured. However, since it is a biotin carboxylase (26), it is likely to use bicarbonate as a substrate (27, 28).

The function of this DIC toolkit has been studied in greatest detail in autotrophs from phylum *Cyanobacteria* and to a lesser extent among a limited number of autotrophic *Proteobacteria* (see below). This understanding of the DIC toolkit is likely to be quite narrow, given that it focuses on organisms from two phyla within domain *Bacteria* using a single pathway (the Calvin-Benson-Bassham cycle). Autotrophy is broadly distributed among multiple phyla of *Archaea* and *Bacteria*, with eight autotrophic DIC fixation pathways known and more likely to be discovered [reviewed in reference (29, 30)]. Besides the Calvin-Benson-Bassham cycle (CBB) (31), there are the reductive citric acid cycle (rTCA) (32), Wood-Ljungdahl pathway (WL) (33), dicarboxylate/4-hydroxybutyrate cycle (DCHB) (34), hydroxypropionate/4-hydroxbutyrate cycle (HPHB) (35), hydroxypropionate bicycle (HP) (36), reverse oxidative citric acid cycle (roTCA) (37, 38), and reductive glycine pathway (39). Our limited understanding of DIC toolkit function, given how critical it is to using DIC as a growth substrate, hinders our understanding of DIC fixation in the many habitats where non-CBB organisms from many phyla catalyze reactions of geochemical importance and contribute to primary productivity. These habitats include the open ocean, sediments and soils, sewage, digestive tracts (e.g., rumen and termite hindguts), terrestrial and marine hot springs, deep-sea hydrothermal vents, and the subsurface (Table S1) (40, 41). Some of these habitats have high CO_2_ concentrations, which could make a DIC toolkit less necessary for autotrophic growth; however, CO_2_ in these habitats can be erratic or low (40, 42–44), and some organisms isolated from them have elaborate DIC toolkits (45). Beyond hamstringing our understanding of primary productivity in a huge variety of habitats, this narrow understanding of DIC toolkit function likely compromises efforts to engineer DIC-fixing organisms and enzymes to enhance crop yields, synthesize compounds of industrial relevance, and incorporate them into carbon-capture technologies (46, 47).

To address this lacuna, this review begins with a description of DIC toolkit components and their function in systems from *Cyanobacteria* and *Proteobacteria* that have been characterized and continues with DIC toolkit presence and predicted function in other phyla based on finished genome sequences from autotrophs using multiple pathways from both *Archaea* and *Bacteria*. The roTCA and reductive glycine pathways are not included because of a lack of marker genes and uncertainties in their taxonomic distributions. *Cyanobacteria* are also excluded from the genome comparisons, as genome surveys of their DIC toolkits have been previously published (48, 49)

## COMPONENTS OF THE DIC TOOLKIT

Carbonic anhydrase (CA) catalyzes the hydration of CO_2_ (forming H_2_CO_3_) and dehydration of H_2_CO_3_ (forming CO_2_). Since the protonation and deprotonation of H_2_CO_3_ are instantaneous, CA activity speeds the interconversion of CO_2_ and HCO_3_^−^, bringing them to chemical equilibrium much more rapidly than in the enzyme’s absence (50). For example, in the enzyme’s absence, under conditions similar to surface seawater (2 mM HCO_3_^−^), the initial rate of CO_2_ production from H_2_CO_3_ is 0.05 mol sec^−1^ L^−1^ (25°C, k_D_ = 26 sec^−1^) (51). This rate would be doubled by adding just 0.6–3 µmol of CA per liter [17–334 mg, based on kinetic parameters from one of the fastest (52) and slowest (53) forms of CA]. Catalyzing this interconversion is beneficial for enzymes using either CO_2_ or HCO_3_^−^ and also facilitates DIC accumulation or dissipation in cells by minimizing diffusive limitation of CO_2_ across membranes (54). As a result, CA is extremely useful to autotrophs and heterotrophs and is ubiquitous among organisms from all three domains of life (55). This ubiquity is accompanied by enzyme diversity. Currently, there are at least six known evolutionarily independent forms of CA: alpha (α) (56), beta (β) (57), gamma (γ) (58), delta (δ) (59), epsilon (ε, CsoSCA; deeply divergent βCA) (53, 60, 61), zeta (ζ, may be deeply divergent βCA) (62), eta (η, may be deeply divergent αCA) (63), theta (θ) (64), and iota (ι) (65). The taxonomic distribution, mechanism, and structure of these enzymes were recently reviewed (6).

DIC transporters are similarly diverse and, among autotrophic prokaryotes, have been described from *Cyanobacteria* and *Proteobacteria*. HCO_3_^−^ transporters from *Cyanobacteria* include three evolutionarily independent forms: SbtA (66) and BicA (a member of the SulP transporter family) (67), which rely on membrane potential for transport, and an ABC transporter (CmpABCD) (68). SbtA-family and SulP-family transporters active on HCO_3_^−^ have also been studied in autotrophic *Proteobacteria*, and a Chr-family transporter was also found to transport HCO_3_^−^ (45). Two evolutionarily distinct types of multisubunit complexes have been described to be active on CO_2_ and facilitate HCO_3_^−^ accumulation in cells. *Cyanobacteria* have two homologous complexes that couple vectoral CA activity (CO_2_ hydrating direction only) to membrane potential via NADH dehydrogenase complexes (69). The second complex, the DIC accumulating complex (DAC), was discovered in *Proteobacteria* and is widespread in many other phyla in *Archaea* and *Bacteria*. It uses extracellular CO_2_ as a substrate to generate elevated intracellular DIC concentrations; the mechanism of this complex remains to be elucidated (70–73).

## DIC TOOLKIT FUNCTION IN AUTOTROPHIC *PROTEOBACTERIA* AND *CYANOBACTERIA* AND PERHAPS ONE AUTOTROPHIC MEMBER OF *BACILLOTA*

The best-studied system with respect to DIC toolkit function is the CO_2_-concentrating mechanism (CCM) present in *Cyanobacteria* and some autotrophic *Proteobacteria*. This system was first suggested in reference (74) and consists of transporters and CA acting in concert to facilitate the growth of cells under low CO_2_ conditions [reviewed in references (75–78)]. Transporters (SbtA, BicA/SulP, and CmpABCD) and CO_2_-active systems [NADH dehydrogenase-associated vectoral CA, or DAC] generate elevated intracellular HCO_3_^−^ concentrations (70, 73, 79). HCO_3_^−^ then enters carboxysomes, which are polyhedral microcompartments with protein shells permeable to HCO_3_^−^ but not CO_2_ (80). Carboxysomes contain ribulose 1,5-bisphosphate carboxylase/oxygenase (RubisCO) and CA (CsoSCA in *Proteobacteria* and some *Cyanobacteria*); carboxysomal CA converts some of the HCO_3_^−^ to CO_2_, which is then fixed by RubisCO [reviewed in reference (81)]. One important aspect of CCM function is the necessity of spatial segregation of HCO_3_^−^ delivery to the cytoplasm from (non-vectoral) CA activity in the carboxysome. Heterologous expression of human CA in the cytoplasm of Cyanobacterium *Synechococcus elongatus* results in loss of the ability to grow under low CO_2_ conditions and massive CO_2_ leakage from cells (82), illustrating that intracellular DIC is not in chemical equilibrium; instead, it is dominated by HCO_3_^−^, which is the form delivered to the cytoplasm by HCO_3_^−^ transporters and CO_2_-active complexes. The presence of extracellular CA has been documented in organisms with CCMs (83, 84), but its role in facilitating DIC uptake in these organisms is unclear.

In *Cyanobacteria*, CCMs are upregulated under low CO_2_ conditions [reviewed in reference (77)]. This is also the case among the limited number of *Proteobacteria* for which CCMs have been studied (45, 85). Some *Proteobacteria* with CCMs also carry genes encoding noncarboxysomal RubisCO. In these organisms, genes encoding carboxysome components and DIC transporters are upregulated under low CO_2_ conditions; under moderate or high CO_2_ conditions, these CCM genes are downregulated, while genes encoding noncarboxysomal RubisCOs are upregulated (45, 86, 87). These noncarboxysomal RubisCOs are very diverse; some are form I enzymes, with large (CbbL) and small (CbbS) subunits (carboxysomal Rubisco is also form I), while others are form II, with a single type of subunit (CbbM), homologous to form I large subunits [reviewed in (88)].

A few studies explore DIC toolkit function beyond CCMs. CA plays a role in DIC supply for some *Proteobacteria* lacking carboxysomes. Facultative CBB autotrophs *Rhodopseudomonas palustris* and *Ralstonia eutropha* (89) both require CA activity to grow under low CO_2_ conditions. For *R. palustris*, this CA activity is extracellular, and likely to facilitate CO_2_ uptake by keeping the periplasmic DIC pool near equilibrium (90). For *R. eutropha*, CA activity is intracellular (89) and presumably functions to provide HCO_3_^−^ for anaplerotic reactions. CA genes are present in many nonoxygenic photoautotrophs, and enzyme activity in some photosynthetic *Alphaproteobacteria* is higher when grown autotrophically (91).

The study of DIC toolkits has been sparse for organisms using pathways besides the CBB cycle. Perhaps, this is because a DIC toolkit seems particularly important to organisms relying on the CBB cycle because of RubisCO’s lack of specificity as a catalyst. RubisCO can use both CO_2_ and O_2_ as substrates (92). When RubisCO acts as an oxygenase, this activity is not productive for cellular growth; cells must regenerate the ribulose 1,5-bisphosphate consumed by the oxygenase reaction using pathways that consume ATP (93). CCMs act to raise the ratio of CO_2_:O_2_ in the cellular microenvironment of RubisCO, favoring the carboxylase activity over oxygenase (92). However, if RubisCO oxygenase activity were the sole factor driving CCM evolution, one would not expect chemolithoautotrophic organisms living in low-O_2_ habitats to have CCMs, but many do (8, 45). This suggests that DIC toolkits should be present beyond CBB autotrophs. The only study available of a possible DIC toolkit in a non-CBB autotroph is one noting the activity of cytoplasmic CA activity in *Acetobacterium woodii* when growing autotrophically, and the authors suggest CA could play a role in facilitating DIC fixation by the WL pathway in this organism (94). Given the widespread nature of CCMs in CBB-using autotrophs from a variety of habitats, some of which co-exist with autotrophs using other pathways [e.g., reference (95)], it seems likely that DIC toolkits are relevant beyond CBB organisms.

## FREQUENCY OF DIC TOOLKIT GENES AMONG GENOMES FROM *BACTERIA* AND *ARCHAEA*

**Fig 1:**
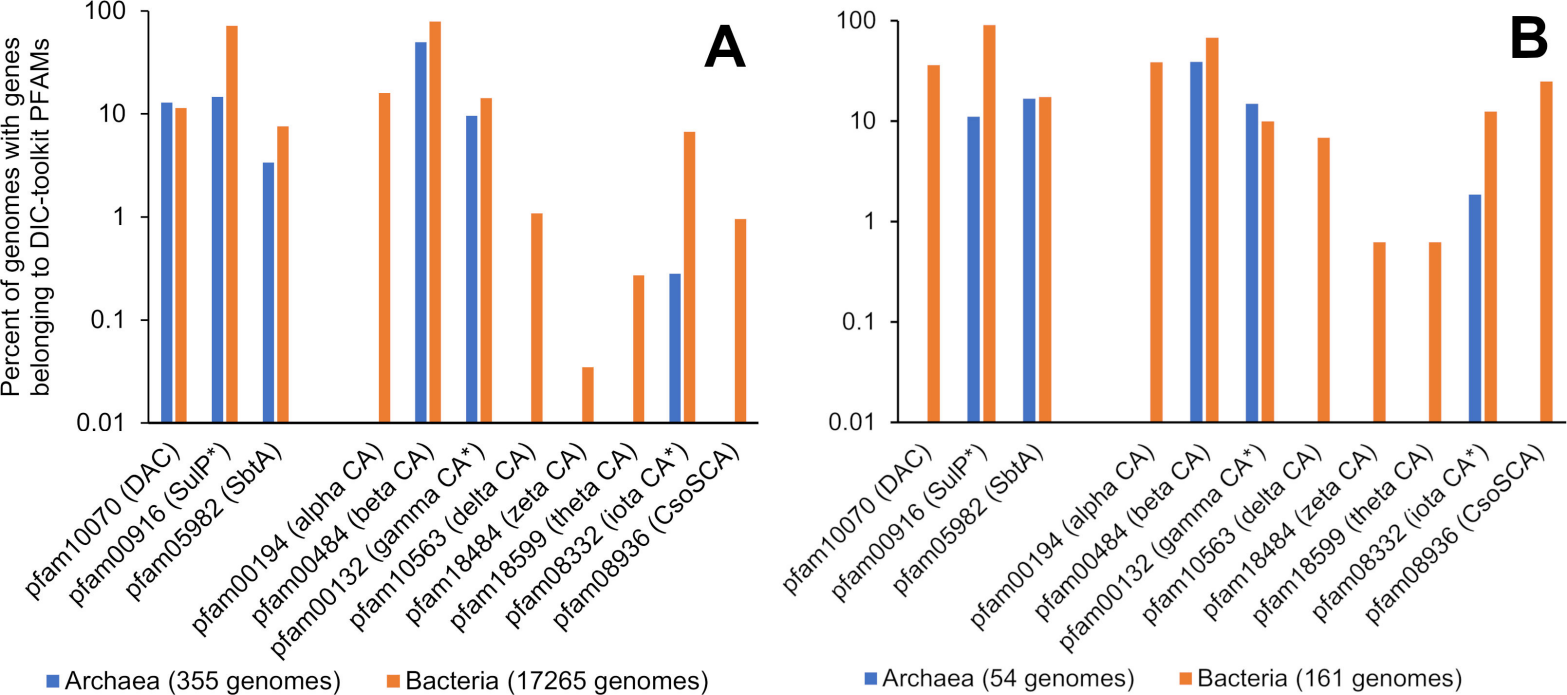
Prevalence of genes encoding DIC transporters (DAC, SulP, and SbtA) and carbonic anhydrase enzymes in finished genomes in the Integrated Microbial Genomes and Microbiomes database (https://img.jgi.doe.gov/) (96). (**A**) Percentage of all finished genomes in IMG with genes belonging to Pfams including DIC transporters and CA. Asterisks indicate Pfams that include members that do not metabolize DIC (Pfam00916 SulP includes sulfate transporters, Pfam00132 includes acyltransferases, and Pfam08332 includes protein kinases). (**B**) Percentage of all finished genomes in IMG from organisms capable of growing autotrophically, with genes belonging to Pfams including DIC transporters and CA. The genomes in B were the ones used for this study and represent organisms capable of fixing DIC via the Calvin-Benson-Bassham cycle, reductive citric acid cycle, Wool-Ljungdal pathway, hydroxypropionate bicycle, dicarboxylate-hydroxybutyrate cycle, or hydroxypropionate-hydroxybutyrate cycle. The procedure used for gathering these genomes is described in Supplemental Material.

Genes likely to encode DIC transporters and CAs are widespread in finished genomes from *Bacteria* and *Archaea* (Fig. 1A). Some toolkit genes are less abundant in *Archaea*, e.g., those encoding some forms of CA (α, δ, ζ, θ, and CsoSCA). Given that new forms of CA continue to be uncovered, the possibility exists that there are novel types of this enzyme that remain to be found. If the genomes are limited to those organisms with a documented ability to grow as autotrophs (Table S1), the level of toolkit gene incidence is higher (Fig. 1B). This is particularly noteworthy, as this smaller sample specifically excludes *Cyanobacteria*, for which the DIC toolkit function has already been extremely well documented (see above). The only gene family that diminishes in abundance is Pfam10070, which includes the cytoplasmic subunits of DACs. This gene family is not present in the autotrophic members of *Archaea* represented in Fig. 1B. DACs are found in members of *Euryarchaeota*, class *Halobacteria* (70, 73); these members are heterotrophs and therefore are not included. Though widespread among both autotrophs and heterotrophs, the fact that toolkit gene abundance is particularly high among autotrophs strongly supports their relevance to autotrophic metabolism.

## EVIDENCE THAT DIC TOOLKIT GENES ARE INVOLVED IN DIC FIXATION IN AUTOTROPHIC *BACTERIA* AND *ARCHAEA*

**Fig 2:**
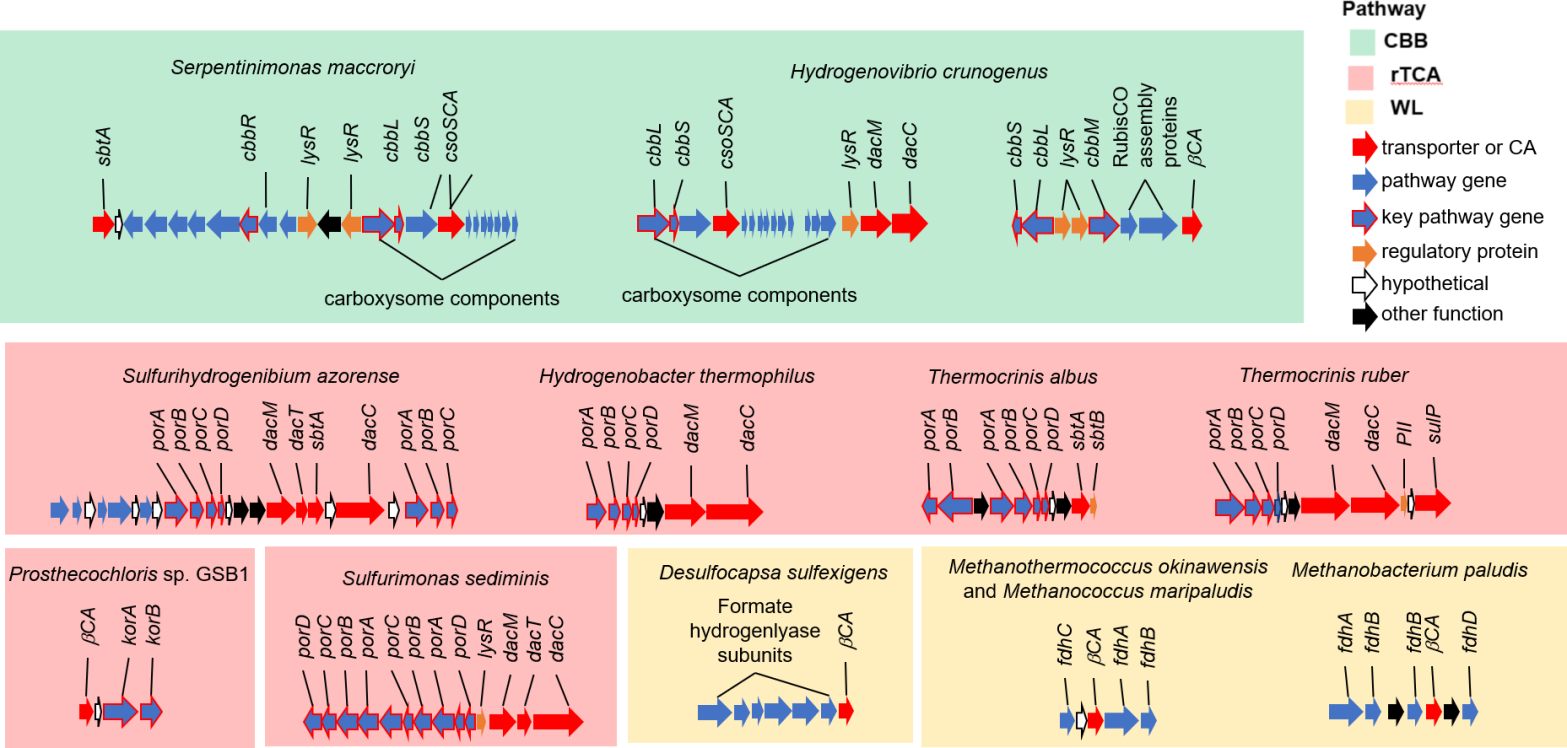
Colocation of genes encoding DIC transporters and carbonic anhydrase with genes from autotrophic DIC fixation pathways. “Pathway genes” encode enzymes catalyzing steps from autotrophic DIC fixation pathways. “Key pathway genes” encode enzymes catalyzing key steps from autotrophic DIC fixation pathways (e.g., CO_2_- or HCO_3_^−^-fixing enzymes: *cbbL*, *cbbS*: form I RubisCO; *cbbM*: form II RubisCO; *porABCD*, *korAB*: pyruvate or 2-oxoglutarate synthase; and *fdhABCD*: formate dehydrogenase).

Prior study has provided many examples of the importance of DIC toolkit genes to autotrophic metabolism; genomic co-location of toolkit genes with those encoding steps of autotrophic DIC fixation pathways provides evidence for yet-to-be-studied connections between toolkit components and DIC fixation. The observation that genes encoding DIC toolkit components neighbor those encoding CBB pathway enzymes has precedence in the literature (8, 97), and only two of the many examples of this co-location are depicted here (Fig. 2). Carboxysome loci include *csoSCA* genes co-located with *cbbL* and *cbbS*, encoding the large and small subunits of carboxysomal form I RubisCO, and also commonly include DIC transporter genes (Fig. 2) (8, 45, 97). Noncarboxysomal RubisCO genes are also sometimes co-located with carbonic anhydrase genes (Fig. 2) (84, 86), which raises the possibility that CA facilitates carbon fixation by RubisCO.

There are some intriguing juxtapositions beyond those anticipated from prior study. Among organisms using the rTCA, DIC transporter or CA genes are co-located with genes encoding enzymes from the rTCA (Fig. 2), suggesting that there are yet-to-be-studied mechanisms for DIC toolkit interactions with this pathway. There is also a recurring juxtaposition in organisms using the WL pathway between genes encoding CA and formate hydrogenlyase or formate dehydrogenase (Fig. 2). It is hard to understand how CA in this context is used by these organisms to facilitate DIC fixation. Formate hydrogenlyase can oxidize formate to CO_2_, while reducing protons to form hydrogen gas (98), though such a capability has yet to be measured in *Desulfocapsa sulfexigens*. Likewise, formate dehydrogenase in methanogenic *Archaea* functions in the formate oxidizing direction to reduce redox cofactor F_420_, which is used primarily as a reductant for methanogenesis and to a minor degree by the WL pathway for cell biosynthesis (99). Instead of facilitating DIC fixation directly, perhaps these CAs facilitate the conversion of CO_2_ produced from formate oxidation to HCO_3_^−^, which in turn could be used by a formate:bicarbonate antiporter to diminish the energetic expense of formate acquisition from the environment by making its acquisition electroneutral.

## PHYLOGENETIC DISTRIBUTION OF DIC TOOLKIT GENES AMONG ORGANISMS CAPABLE OF AUTOTROPHIC GROWTH USING DIFFERENT PATHWAYS

**Fig 3:**
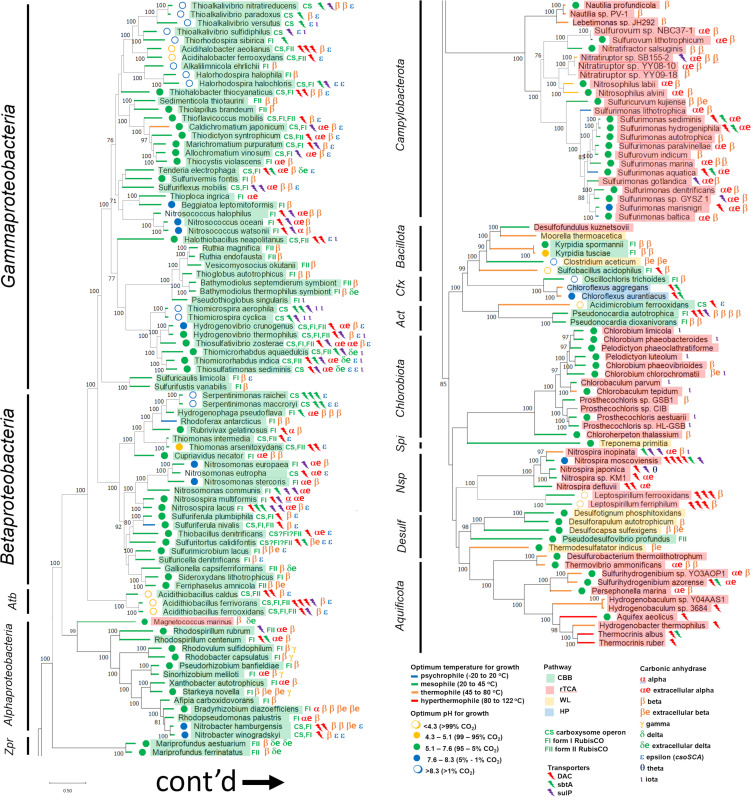
Taxonomic distribution of genes encoding DIC transporters (DAC, SulP, and SbtA) and carbonic anhydrase enzymes among members of *Bacteria*. Optimum growth conditions and autotrophic DIC fixation pathway are also provided. Maximum likelihood trees are based on concatenated alignments of amino acid sequences predicted from genes encoding ribosomal proteins. Genes were gathered, aligned, and concatenated from Ribosomal MLST [https://pubmlst.org/species-id (100)]). This alignment of 9,939 positions was used to generate a maximum likelihood tree in MEGA 11 (101) after finding the best model [Le-Gascuel (102), gamma distribution (five categories), and invariant sites]. Bootstrap values are based on 100 resamplings of the alignment. Phyla and classes were gathered from https://lpsn.dsmz.de/, the List of Prokaryotic names with Standing in Nomenclature, with the following exceptions: “*Desulfobacterota*” are based on reference (103), and *Candidatus* Zetaproteobacteria are based on (104). Autotrophic pathways were inferred from genome sequences and the literature, and optimum pH and temperatures for growth were gathered from the literature as well (Table S1). Predicted functions for gene products from genes encoding potential DIC transporters or carbonic anhydrase enzymes were verified using predictions of transmembrane helices (transporters) and conserved residues (carbonic anhydrase) as described in Table S2. Extracellular locations for carbonic anhydrase enzymes were predicted using SignalP 6.0 (https://services.healthtech.dtu.dk/services/SignalP-6.0/) (105). The fraction of DIC present as CO_2_ at optimal growth pH, when available, was calculated using pK_1_ = 6.35 and pK_2_ = 10.33 (2). *Act*, *Actinomycetota*; *Atb*, *Acidithiobacillia*; *Cfx*, *Chloroflexota*; *Desulf*, “*Desulfobacterota*”; *Nsp*, “*Nitrospirae*;” *Spi*, *Spirochaetota*; *Zpr*, *Candidatus* Zetaproteobacteria; CBB, Calvin-Benson-Bassham cycle; HP, hydroxypropionate bicycle; rTCA, reductive citric acid cycle; WL, Wood-Ljungdal pathway.

**Fig 4:**
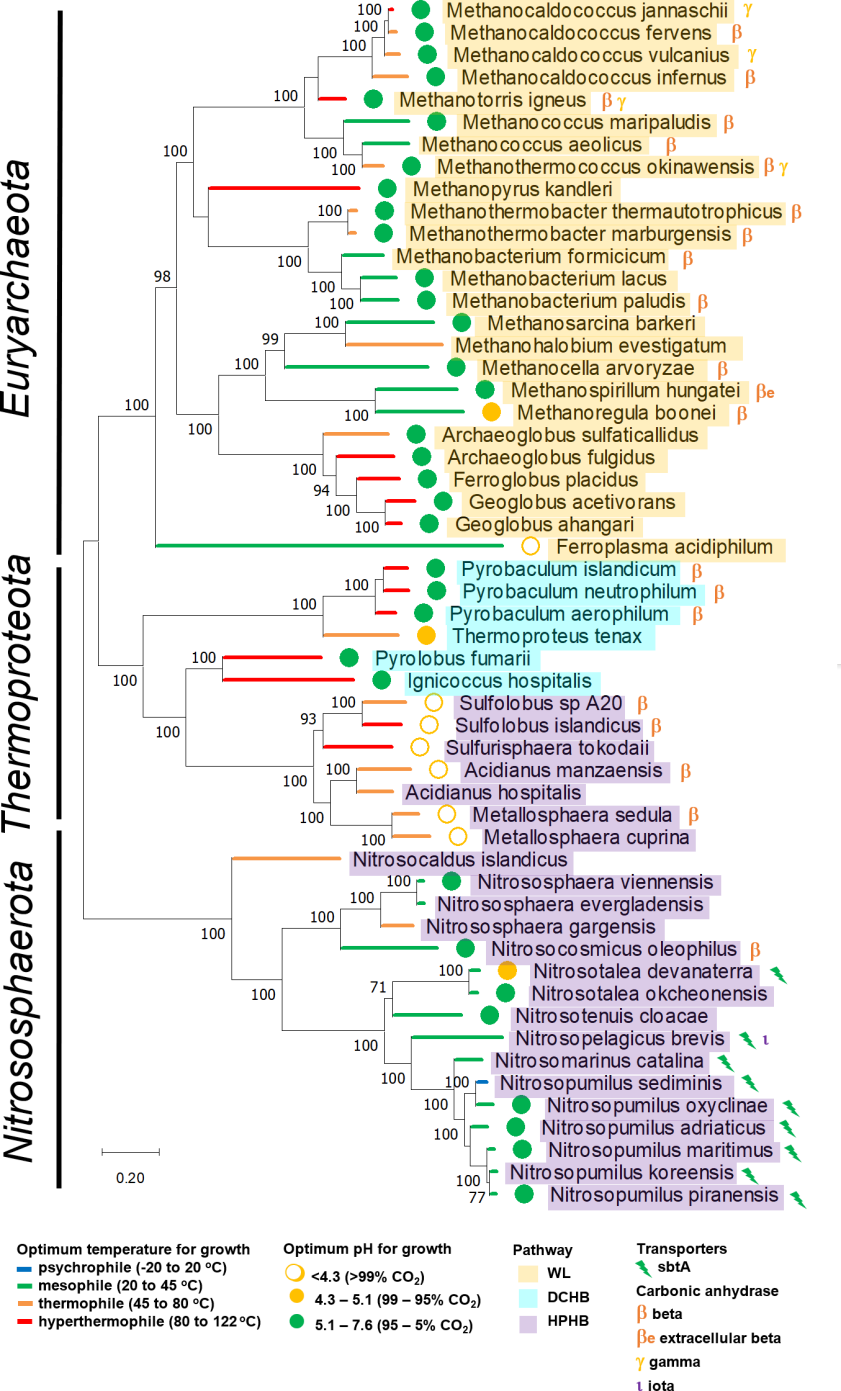
Taxonomic distribution of genes encoding DIC transporters (DAC, SulP, and SbtA) and carbonic anhydrase enzymes among members of *Archaea*. Optimum growth conditions and autotrophic DIC fixation pathway are also provided. Maximum likelihood trees are based on concatenated alignments of amino acid sequences predicted from genes encoding ribosomal proteins. Genes were gathered from genome sequences using COGs comprised of ribosomal large and small subunits. Amino acid sequences predicted from genes encoding each subunit were aligned via MUSCLE (MUltiple Sequence Comparison by Log-Expectation) (106) and concatenated using a script available from https://github.com/scooterboi85/Gene-concatenator, resulting in an alignment of 8,612 positions. Maximum likelihood analysis was implemented as described in Fig. 3. Phyla and classes were gathered from https://lpsn.dsmz.de/, the List of Prokaryotic names with Standing in Nomenclature. Autotrophic pathways were inferred from genome sequences and the literature, and optimum pH and temperatures for growth were gathered from the literature as well (Table S1). For members of genus *Pyrobaculum*, genome data suggest the DCHB pathway, but other evidence is less conclusive (107, 108). Predicted functions and cellular locations for gene products from genes encoding potential DIC transporters or carbonic anhydrase enzymes were verified as described in Fig. 3; Table S2. The fraction of DIC present as CO_2_ at optimal growth pH, when available, was calculated using pK_1_ = 6.35 and pK_2_ = 10.33 (2). DCHB, dicarboxylate-hydroxybutyrate cycle; HPHB, hydroxypropionate-hydroxybutyrate cycle; WL, Wood-Ljungdal pathway.

The CBB, rTCA, WL, and HPHB are well represented among autotrophic organisms with finished genomes, while the HP and DCHB are much less so (Fig. 3 and 4). DIC toolkit genes are very broadly taxonomically distributed in autotrophic *Bacteria* (9 out of 10 phyla) and *Archaea* (all 3 phyla). Given that this sampling only includes finished genomes, which are a minority of sequenced genomes (~12% as of 4 August 2023; https://img.jgi.doe.gov/), it is likely that these genes are present in autotrophs from many other phyla. The toolkit is particularly well represented in organisms using the CBB in phyla *Proteobacteria*, *Bacillota*, and *Actinomycetota*, as well as organisms using the rTCA in phyla *Campylobacterota* and *Aquificota* and those using the HP in *Chloroflexota*.

Toolkit genes are less abundant among autotrophic *Archaea* (Fig. 4). βCA and γCA are represented among the phyla, while SbtA transporters are present in some members of *Nitrososphaerota*. Given the relative abundance of toolkit components in autotrophic *Bacteria* and the recent discoveries of novel forms of CA (65) and DIC accumulation (70–73), it seems likely that this paucity reflects the fact that domain *Archaea* is comparatively understudied.

Patterns of gene presence and absence sometimes follow organism taxonomy (Fig. 3 and 4). For example, all members of the *Ruthia/Vesicomyosocius/Thioglobus/Bathymodiolus* symbiont clade lack DIC transporter genes and carry βCA genes (Fig. 3). However, there are many departures from taxonomy. There is within-genus divergence. Both members of *Hydrogenovibrio* have genes encoding DAC, CsoSCA, and βCA, but genes encoding SulP, αCA, and ιCA are not present in both. Rather, extreme divergence is apparent within genus *Pseudonocardia*; both members of this genus carry genes encoding βCA, but one member (*autotrophica*) carries three DIC transporter genes, while the other (*dioxanivorans*) has none. The autotrophic DIC-fixing pathway and environment appear to play a role in DIC toolkit distribution. Organisms using the HPHB appear to have toolkit components that correlate with their optimal pH for growth, while organisms from multiple phyla using the CBB or rTCA are particularly “loaded,” suggesting DIC toolkit distribution might be convergent with the autotrophic DIC fixation pathway (Fig. 3 and 4). Accordingly, the following sections explore the correlation between DIC toolkit components and the environment and autotrophic pathway.

## DISTRIBUTION OF DIC TOOLKIT GENES RELATIVE TO ENVIRONMENTAL DIC SUPPLY

Autotrophs in this study have optimal pH values for growth ranging from 1.4 to 11 (Table S1) and therefore thrive in environments with dramatic differences in DIC composition. DIC composition is sensitive to pH, with CO_2_ dominating below the pK_1_ for carbonic acid (~pH 6.4), CO_3_^=^ dominating above the pK_2_ (~10.3), and HCO_3_^−^ dominating at circumneutral pH (2), where cytoplasmic pH is poised, even in acidophilic and alkaliphilic microorganisms (109, 110).

**Fig 5:**
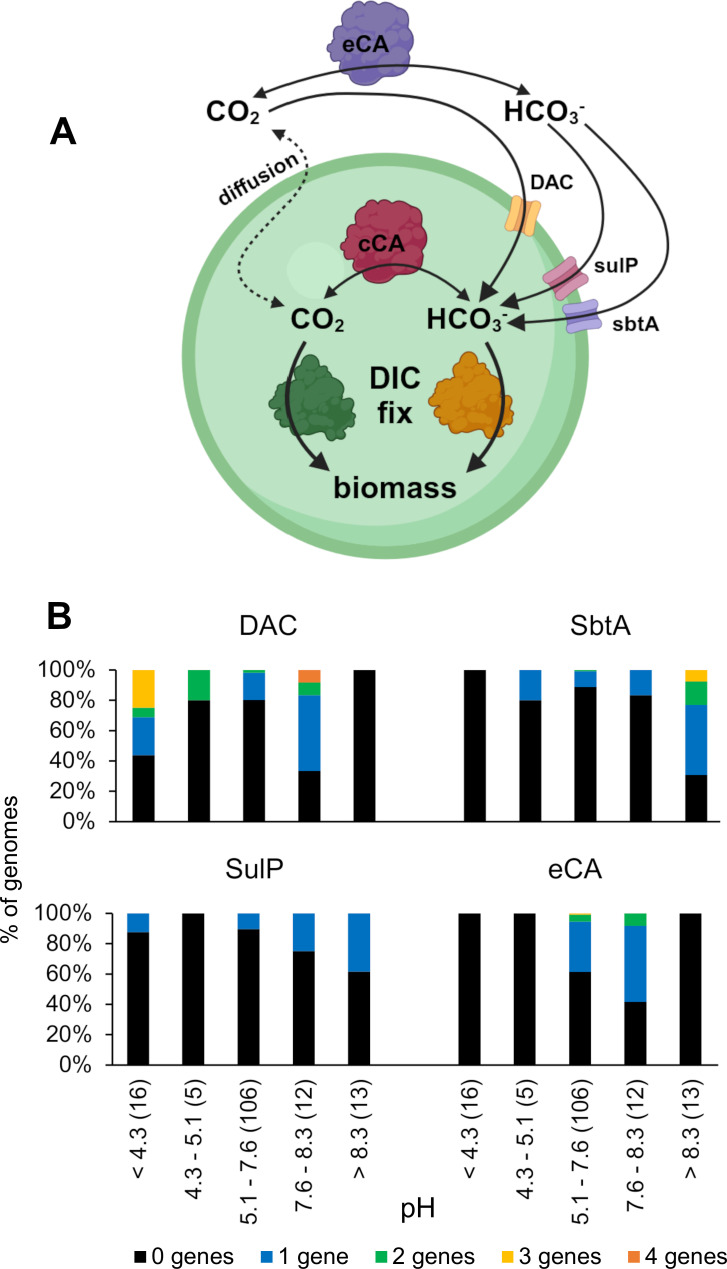
Potential functioning of DIC toolkit components within a cell and with the environment. (**A**) General model of an autotrophic cell, showing the location of DIC toolkit components. (**B**) Trends in DIC toolkit component presence and abundance with optimum growth pH for the host organism. pH ranges were chosen to reflect the following % of DIC that is in the form of CO_2_: <4.3: at least 99% CO_2_; 4.3–5.1: 99 – 95% CO_2_; 5.1–7.6: 95 – 5% CO_2_; 7.6–8.3: 5 – 1% CO_2_; and >8.3: less than 1% CO_2_. Numbers in parentheses are the numbers of genomes in each category. cCA, cytoplasmic carbonic anhydrase; DAC, DIC accumulating complex; DIC fix, DIC fixation; eCA, extracellular carbonic anhydrase; SbtA, SbtA family transporter; SulP, SulP family transporter.

The ways in which CAs and DIC transporters could potentially facilitate growth in environments with differing DIC compositions is illustrated in Fig. 5A. DIC transporters using CO_2_ or HCO_3_^−^ could facilitate uptake at different environmental pH values, and extracellular CA could prevent the concentration of CO_2_ or HCO_3_^−^ from dropping below equilibrium values if consumed by the cell.

The presence of DIC toolkit components does correlate with pH (Fig. 5B). The distribution of different DIC transporters does seem to follow the environmental abundance of the form of DIC transported: DACs are absent in organisms with pH optima above 8.3 and SbtA transporters are absent in organisms with pH optima below 4.3, conditions where their substrates (CO_2_ or HCO_3_^−^, respectively) are less than 1% of DIC. This trend mirrors what has been observed in metagenomes (73). Similar to SbtA, genes encoding SulP transporters likely to be active on HCO_3_^−^ are more abundant in organisms growing at high pH (Fig. 5B). The ability to transport HCO_3_^−^ by this type of transporter was predicted by phylogenetic analysis (Fig. S1); these predictions would be stronger if more SulP family transporters were biochemically characterized, since these transporters are active on a variety of compounds (111, 112).

Genes predicted to encode extracellular CA are absent from organisms growing below pH 5.1 or above 8.3 (Fig. 5B). One possibility is that this distribution indicates that these enzymes are pH labile. Another possibility is that these enzymes would not be particularly helpful at extremely acidic or alkaline pH; their ability to bring DIC to equilibrium would not facilitate CO_2_ or HCO_3_^−^ supply when taking place at pH values where either CO_2_ or HCO_3_^−^ are extremely scarce at equilibrium.

## CYTOPLASMIC CO_2_ AND HCO_3_^−^ DEMAND BY DIFFERENT AUTOTROPHIC PATHWAYS

**Fig 6:**
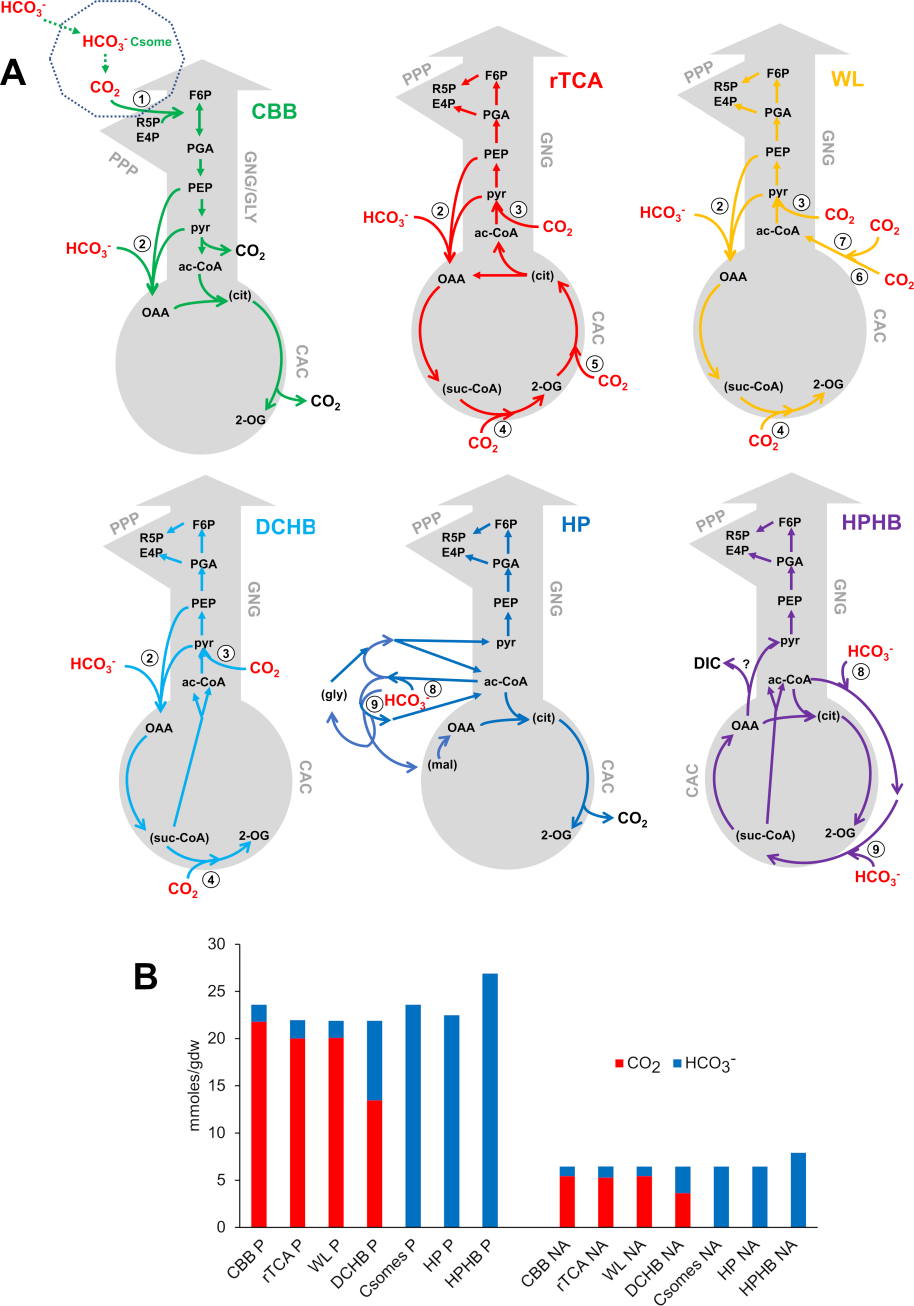
CO_2_ and HCO_3_^−^ consumption by organisms using different autotrophic DIC fixation pathways. (**A**) Overview of steps from autotrophic DIC fixation to the synthesis of metabolic intermediates necessary for protein and nucleotide biosyntheses. Some metabolic intermediates that are not themselves utilized for biosynthesis have been added for clarity and are enclosed in parentheses. Forms of DIC fixed by enzymes catalyzing autotrophic DIC fixation are from the references cited in Table 1. For CBB, rTCA, WL, and DCHB, arrows from both PEP and pyr reflect the variable distributions of phosphoenolpyruvate carboxylase and pyruvate carboxylase among *Bacteria* and *Archaea* (23, 113, 114). (**B**) Amounts of CO_2_ and HCO_3_^−^ necessary to synthesize protein (P) or nucleic acids (NA) for 1-gram dry weight of biomass of a generic cell using different DIC fixation pathways. An approach similar to (115) was used to calculate the contribution of CO_2_ and HCO_3_^−^ to the synthesis of macromolecules in autotrophic organisms using different autotrophic DIC fixation pathways (Supplemental Material). The mmoles of DIC consumed are greater for organisms using the CBB, Csomes, HP, and HPHB pathways due to losses during synthesis of metabolic intermediates (**A**) 1, RubisCO; 2, phosphoenolpyruvate carboxylase or pyruvate carboxylase; 3, pyruvate synthase; 4, 2-oxoglutarate synthase; 5, isocitrate dehydrogenase; 6, formate dehydrogenase (*Bacteria*) or formylmethanofuran dehydrogenase (*Archaea*); 7, carbon monoxide dehydrogenase/acetyl-CoA synthase; 8, 9, acetyl-CoA/propionyl-CoA carboxylase; ac-CoA, acetyl-coenzyme A; CAC, citric acid cycle; CBB, Calvin-Benson-Bassham cycle; cit, citrate; Csomes, carboxysome; DCHB, dicarboxylate-hydroxybutyrate cycle; DIC, dissolved inorganic carbon; E4P, erythrose 4-phosphate; F6P, fructose 6-phosphate; GNG/GLY, gluconeogenesis/glycolysis; gly, glyoxylate; HP, hydroxypropionate bicycle; HPHB, hydroxypropionate-hydroxybutyrate cycle; mal, malate; OAA, oxaloacetate; 2-OG, 2-oxoglutarate; PEP, phosphoenolpyruvate; PGA, 3-phosphoglyceraldehyde; PPP, pentose phosphate pathway; pyr, pyruvate; R5P, ribose 5-phosphate; rTCA, reductive citric acid cycle; suc-CoA, succinyl-coenzyme A; WL, Wood-Ljungdahl pathway.

Some of the carboxylases catalyzing autotrophic, anaplerotic, and biosynthetic DIC fixation use CO_2_ as a substrate, while others use HCO_3_^−^ (Table 1). As a result, organisms using different autotrophic DIC-fixing pathways have differing demands for cytoplasmic CO_2_ and HCO_3_^−^ for synthesizing the metabolic intermediates necessary for generating biomass (Fig. 6). Pathways which predominantly incorporate CO_2_ into biomass include CBB, rTCA, and WL, though they also require HCO_3_^−^ for oxaloacetate synthesis likely by phosphoenolpyruvate carboxylase or pyruvate carboxylase (Table 1; Fig. 6A). The contributions of CO_2_ and HCO_3_^−^ to the biomass of DCHB autotrophs are more evenly split, while HCO_3_^−^ is the dominant form of DIC incorporated by organisms using the HP and HPHB pathways. HCO_3_^−^ is also the dominant form of DIC incorporated by organisms with carboxysomes, even though they use the CBB cycle. In these organisms, RubisCO draws from the pool of CO_2_ present in carboxysomes, which originated from cytoplasmic HCO_3_^−^ that was dehydrated by carboxysomal CA after entering carboxysomes (74, 80). For organisms whose genomes encode both carboxysomal as well as noncarboxysomal RubisCO, the contributions of HCO_3_^−^ and CO_2_ to biomass will depend on whether the cells are growing under conditions when carboxysome synthesis is induced (e.g., low CO_2_) or when noncarboxysomal RubisCO is predominant (e.g., high CO_2_).

## DISTRIBUTION OF DIC TOOLKIT GENES IN AUTOTROPHIC ORGANISMS RELYING PRIMARILY ON CO_2_

**Fig 7:**
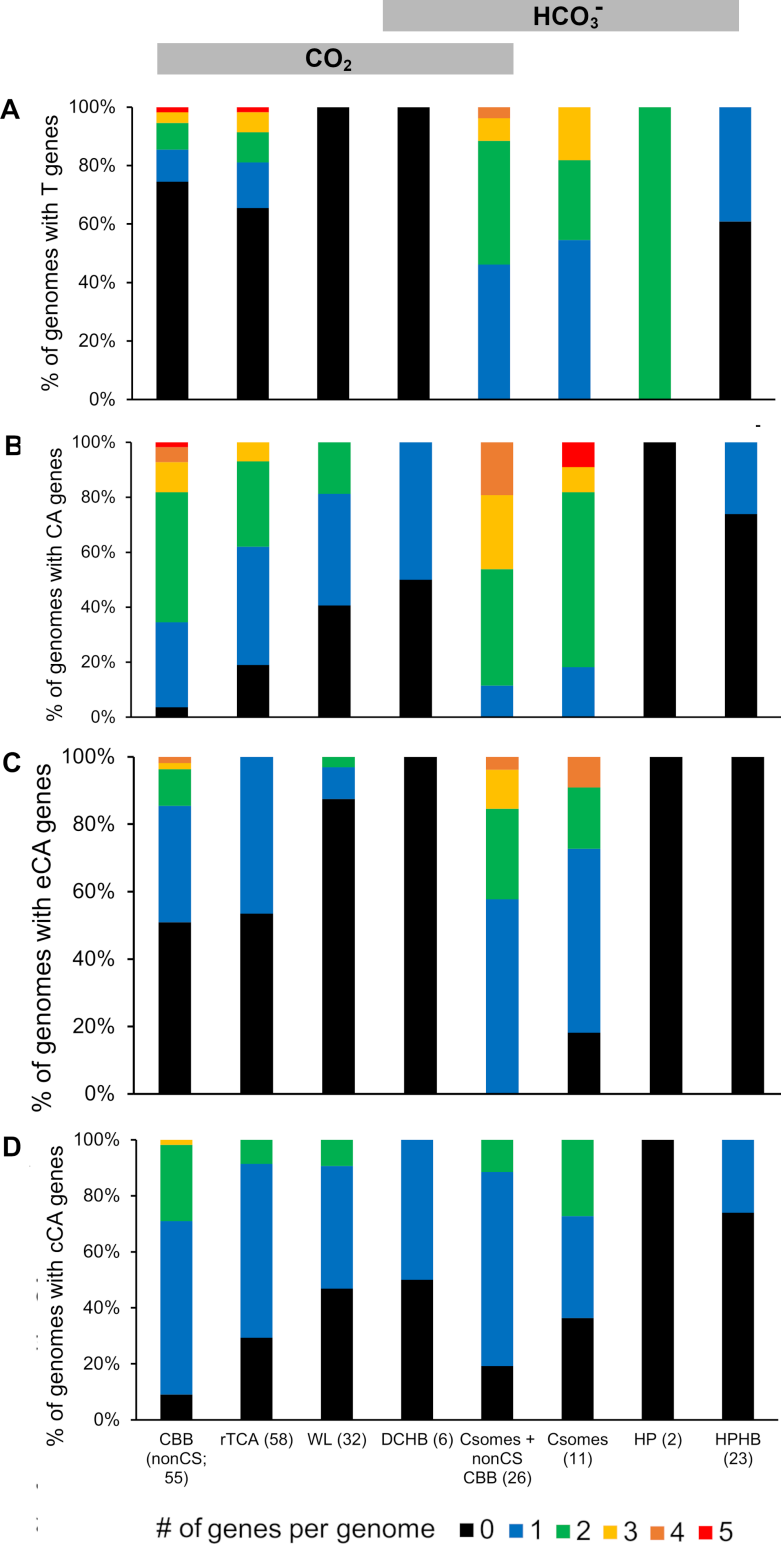
Number of genes encoding DIC transporters or carbonic anhydrase per genome in organisms using different autotrophic DIC fixation pathways. Pathways are positioned according to Fig. 6, with those relying predominantly on CO_2_ toward the left, and those relying predominantly on HCO_3_^−^ toward the right. Pathways are listed with the number (N) of genomes representing them. (**A**) Number of DIC transporter genes (T) per genome; (**B**) number of CA genes per genome (includes *csoSCA*); (**C**) number of extracellular CA genes (eCA) per genome; (**D**) number of cytoplasmic CA genes (cCA) per genome (excludes *csoSCA*). For both C and D, the CA location was predicted by SignalP 6.0 (https://services.healthtech.dtu.dk/services/SignalP-6.0/) (105). CBB (nonCS), genomes encoding the CBB cycle, with only noncarboxysomal form I or form II RubisCO; rTCA, reductive citric acid cycle; WL, Wood-Ljungdahl pathway; DCHB, dicarboxylate-hydroxybutyrate cycle; Csomes + nonCS CBB, genomes encoding carboxysomes as well as noncarboxysomal form I and/or form II RubisCO; Csomes, genomes encoding carboxysomes, lacking noncarboxysomal RubisCO; HP, hydroxypropionate bicycle; HPHB, hydroxypropionate-hydroxybutyrate cycle.

**Fig 8:**
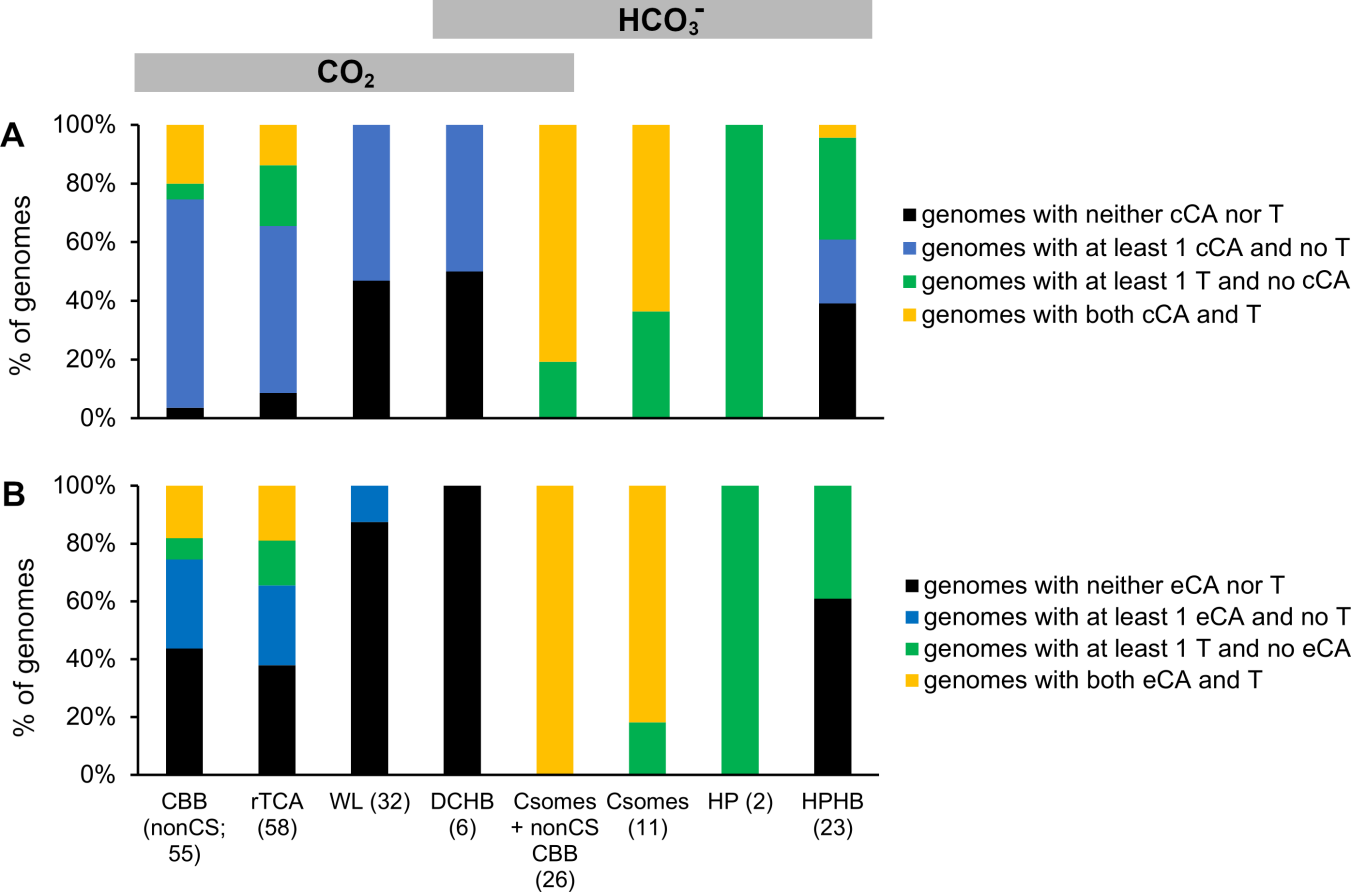
Coexistence of potential DIC transporter genes (T) and (A) cytoplasmic CA genes or (B) extracellular CA genes within genomes from organisms with different autotrophic DIC fixation pathways. Numbers in parentheses are the numbers of genomes in each category. The CA location was predicted by SignalP 6.0 (https://services.healthtech.dtu.dk/services/SignalP-6.0/) (105). eCA, extracellular carbonic anhydrase; cCA, cytoplasmic carbonic anhydrase; CBB (nonCS), genomes encoding the CBB cycle, with only noncarboxysomal form I or form II RubisCO; rTCA, reductive citric acid cycle; WL, Wood-Ljungdahl pathway; DCHB, dicarboxylate-hydroxybutyrate cycle; Csomes + nonCS CBB, genomes encoding carboxysomes as well as noncarboxysomal form I and/or form II RubisCO; Csomes, genomes encoding carboxysomes, lacking noncarboxysomal RubisCO; HP, hydroxypropionate bicycle; HPHB, hydroxypropionate-hydroxybutyrate cycle.

Given the large differences in the demand for CO_2_ and HCO_3_^−^ predicted for organisms using different autotrophic DIC fixation pathways, it is not surprising that organisms using them have large differences in DIC toolkits (Fig. 7). For organisms relying primarily on CO_2_ (CBB, rTCA, and WL), genes encoding DIC transporters are less abundant than for those organisms relying primarily on HCO_3_^−^.(Fig. 7A). Genes encoding CA are quite common and vary among the pathways (Fig. 7B through D). For cells without DIC transporters, provided that environmental pH is not alkaline enough to make extracellular CO_2_ scarce, CO_2_ can diffuse into cells through the membranes or aquaporins before fixation by CO_2_-requiring carboxylases in the cytoplasm. However, these cells also require some HCO_3_^−^ for oxaloacetate and pyrimidine synthesis, which could be provided by either cytoplasmic CA (cCA) from intracellular CO_2_ or DIC transporters from extracellular DIC (Fig. 5A). Indeed, most organisms relying primarily on CO_2_ have genes encoding either cCA or DIC transporters (Fig. 8). Some have genes encoding both, which could be a conundrum.

The simultaneous presence of both cCA and DIC transporters is problematic, as cCA would convert cytoplasmic HCO_3_^−^ delivered by transporters into CO_2_, which would diffuse out of the cell, dissipating the electrochemical gradients that DIC transporters couple to HCO_3_^−^ acquisition (45, 66–68, 70–73). Indeed, when *Cyanobacteria* with DIC transporters are engineered to express cCA, massive amounts of CO_2_ diffuse out of them (82). Perhaps organisms whose genomes encode both cCA and DIC transporters differentially express them, so that they are not present simultaneously. However, recent models indicate that low to moderate levels of co-expression of cCA and DIC transporters can facilitate the simultaneous supply of CO_2_ and HCO_3_^−^ for biosynthesis without CO_2_ leakage (116).

Interestingly, 10 of the 14 CBB and rTCA organisms with genes encoding both DIC transporters and cCA are likely exposed to N_2_O gas during growth. Six use ammonia as their electron donor (and produce N_2_O as a by-product) (117), three use nitrite as an electron donor and likely are exposed to N_2_O produced by the ammonia-oxidizing microorganisms with which they commonly co-occur (118), and one uses N_2_O as an electron donor. Given that N_2_O molecules are similar in size and shape to CO_2_, perhaps, CO_2_-dependent carboxylases and potentially also CA (but not αCA) (119) in these organisms are sensitive to this dissolved gas and the additional DIC toolkit compensates for this inhibition by increasing the concentration of cytoplasmic CO_2_, which could mitigate competitive inhibition by N_2_O.

Organisms using the CBB are generously endowed with genes encoding CA, and many of these are predicted to be extracellular, though almost all of these organisms are predicted to have cytoplasmic CA (Fig. 7B through D). Some of these organisms are intracellular chemolithoautotrophic symbionts of bivalves (*Ruthia magnifica* and *endofausta*, *Vesicomyosocius okutanii*, *Bathymodiolus septemdierum* and *thermophilus* symbionts) and lack DIC transporter genes. Their lack of DIC transporters and reliance on cCA for bicarbonate could be an adaptation to living in the high CO_2_ habitat within actively metabolizing eukaryotic cells. For those organisms using the CBB that have DIC transporter genes, the majority also have eCA genes (Fig. 8). If coexpressed, the eCA could facilitate transporter activity as described above.

Organisms using the rTCA have DIC transporter and CA gene frequencies similar to those using CBB (Fig. 7 and 8); perhaps, this reflects their similarities in demand for CO_2_ and HCO_3_^−^ (Fig. 6). Interestingly, there does appear to be a bimodal distribution of DIC toolkit genes among organisms using the rTCA. Most of the organisms from phylum *Chlorobiota* encode a single carbonic anhydrase and no DIC transporters, while those from phyla *Campylobacterota*, “*Nitrospirae*,” and *Aquificota* typically encode multiple CAs, at least one DIC transporter, or both (Fig. 3). This bimodal distribution of DIC toolkit genes may suggest that some organisms using the rTCA are adapted to lower CO_2_ habitats (*Campylobacterota*, “*Nitrospirae*,” and *Aquificota*) and others to higher (*Chlorobiota*), analogous to low CO_2_-adapted (with carboxysomes) and higher CO_2_-adapted (without carboxysomes) organisms using the CBB. Adaptation to low vs. high CO_2_ habitats in organisms using the rTCA is also supported by their predicted mechanism for aminoimidazole ribonucleotide (AIR) carboxylation in purine biosynthesis. In most *Bacteria* and *Archaea*, two enzymes [5-(carboxyamino)imidazole ribonucleotide synthase, EC 6.3.4.18, encoded by *purK*, and N (5)-carboxyaminoimidazole ribonucleotide mutase, EC 5.4.99.18, encoded by *purE*] act together to carboxylate AIR (120)) PurK uses HCO_3_^−^ as a substrate and passes it to PurE (121). When DIC concentrations are very high, PurE can carboxylate AIR in the absence of PurK, using CO_2_ (122). Consistent with this observation, *Cyanobacteria* with mutations in *purK* require high CO_2_ concentrations for growth (123). Many members of *Chlorobiota* only encode PurE; it is possible that AIR carboxylation is via CO_2_ in these members of *Chlorobiota* (124), and as a result, their growth may require high CO_2_ concentrations. Similar to PurK assisting PurE, a biotin carboxylase is present some members of *Aquificota* that assists isocitrate dehydrogenase by catalyzing the carboxylation of 2-oxoglutarate via HCO_3_^−^. In its absence, isocitrate dehydrogenase uses CO_2_ (Table 1) (12). The biotin carboxylase could facilitate growth under low CO_2_ conditions by diminishing the demand for intracellular CO_2_.

*Archaea* and *Bacteria* using the WL pathway completely lack DIC transporters and have fewer genes encoding CA than CBB or rTCA organisms, though the cCA gene presence and number are similar to rTCA (Fig. 7). It is possible that organisms using the WL pathway are adapted to particularly high CO_2_ habitats, which is also consistent with the majority of them having a purine biosynthetic pathway that requires high CO_2_ (*purE*; see above). Indeed, the acetogens included in this group do require elevated CO_2_ for growth (94). However, the absence of known DIC transporters does not rule out novel DIC transporters; it has been suggested that these organisms may use a yet-to-be characterized acetate-HCO_3_^−^ antiporter (94).

## DISTRIBUTION OF DIC TOOLKIT GENES IN AUTOTROPHIC ORGANISMS RELYING PRIMARILY ON HCO_3_^−^

In general, genes encoding DIC transporters are particularly abundant among autotrophs with DIC fixation pathways relying primarily on HCO_3_^−^ (Fig. 7A), though HPHB organisms have fewer DIC transporter genes than the other HCO_3_^−^-dependent autotrophs. However, among fellow members of *Archaea*, HPHB organisms have more DIC transporter genes than CO_2_-dependent WL and DCHB organisms do (Fig. 7). The relative abundance of DIC transporters among autotrophs that predominantly fix HCO_3_^−^ is particularly sensible, since the HCO_3_^−^ that the transporters deposit in the cytoplasm could be used directly for HCO_3_^−^ fixation. The abundance of cCA is broadly similar to CO_2_-dependent autotrophs, though the HP and HPHB autotrophs have fewer than the others (Fig. 7D). The relative scarcity of cCA genes in HP and HPHB organisms would diminish loss of the cytoplasmic HCO_3_^−^ pool that their transporters had delivered; the presence of cytoplasmic CA would convert a portion of this pool to CO_2_, which could be lost by diffusion through the membrane (3).

Organisms with carboxysomal loci are very generously equipped with both DIC transporter genes and CA (Fig. 7). This observation is consistent with the model of CCM function constructed for *Cyanobacteria*, as described above. Many of these abundant CA genes are predicted to encode extracellular enzymes (Fig. 7C), which may function to supply HCO_3_^−^ or CO_2_ to DIC transporters. This would be particularly helpful if transporter activity is high enough to bring HCO_3_^−^ or CO_2_ concentrations below those present at equilibrium. The number of cCA genes (Fig. 7D) is similar to other organisms, which is a bit alarming, since carboxysomal carbonic anhydrase CsoSCA was not included in this tally, and cCA presents a risk to these cells by facilitating cytoplasmic HCO_3_^−^ leakage by converting it to CO_2_. Perhaps, some of these cCAs have been incorrectly assigned to the cytoplasm by SignalP 6.0 (https://services.healthtech.dtu.dk/services/SignalP-6.0/)(105).

The two HP organisms have genes encoding both DAC and SbtA DIC transporters and an absence of CA genes of any sort, which is quite interesting since the majority of the other organisms do have CA genes. An absence of CA, and presence of DIC transporter genes, is completely consistent with HCO_3_^−^ use by the HP cycle (Fig. 6A). Given the small sample size (two finished genomes), it is not possible to know if this is typical for organisms using this pathway for autotrophic DIC fixation.

Genomes from organisms using the HPHB are the only members of *Archaea* in this study to have genes encoding DIC transporters (SbtA; Fig. 4 and 7). CA gene abundance is similar to other members of *Archaea* (Fig. 7B) and is predicted to be cytoplasmic (Fig. 7D). HPHB organisms with DIC transporters tend not to have cCA and vice-versa, though one organism does have both (Fig. 8A). This pattern of one-or-the-other (cCA vs. DIC transporter) for HCO_3_^−^ supply is similar to what has been observed in *Firmicutes* (125), which minimizes leakage losses expected if both are highly expressed. Additionally, in this case, there appears to be an environmental component. Eight of nine of the HPHB organisms that have DIC transporter genes grow optimally at circumneutral pH, while four of five that have cCA are acidophilic (Fig. 4). SbtA transport requires HCO_3_^−^, which is not present at acidic pH, so *sbtA* gene absence from most of the acidophiles makes sense. Likewise, reliance on a cCA for cytoplasmic HCO_3_^−^ in turn relies on diffusion of CO_2_ from the environment, which is a better strategy in acidic environments than circumneutral ones, where the proportion of DIC as CO_2_ is lower. The presence of both a DIC transporter and cCA gene in *Nitrosopelagicus brevis* is curious, as it is for the other organisms using other pathways.

## DISTRIBUTION OF DIC TOOLKIT GENES IN AUTOTROPHIC ORGANISMS RELYING ON CO_2_ AND HCO_3_^−^

Organisms using the DCHB, which requires nearly equal amounts of CO_2_ and HCO_3_^−^ simultaneously (Fig. 6), do not have genes encoding known DIC transporters, and half have genes encoding cCA (Fig. 7). Those with cCA belong to genus *Pyrobaculum*. Though genome data suggest these organisms use DCHB (107), biochemical data are less conclusive, suggesting the rTCA could operate in these organisms (108). In this case, the presence of a different toolkit could reflect the use of a different pathway. The relative paucity of DIC toolkit genes may reflect the comparatively understudied nature of *Archaea*. If this paucity indeed reflects the actual abundance of DIC toolkit genes in these organisms, the ones with cCA are relying on diffusion of CO_2_ from the environment for cytoplasmic CO_2_ and (cCA-mediated) HCO_3_^−^ supply. The organisms lacking both cCA and DIC transporters raise another possibility. Most of these organisms are hyperthermophiles (five of six); the remaining one is a thermophile (Fig. 4). All were isolated from hot springs (Table S1). Given that membrane permeability to CO_2_ (126) and chemical (non-CA) DIC interconversion rates (2) both increase with temperature, perhaps, a DIC toolkit is less necessary for these organisms. However, it is important to note that thermophiles and hyperthermophiles using other pathways do have DIC toolkit components, including DIC transporters (e.g., members of phylum *Aquificota;* Fig. 3). The presence of DIC transporter genes in thermophilic and hyperthermophilic *Bacteria* suggests that transporters could be helpful for thermophilic and hyperthermophilic *Archaea*, especially since their cell membrane permeabilities have been found to be less sensitive to temperature than those present from hyperthermophilic *Bacteria* (127). Taken together, these observation suggest that *Archaea* using the DCHB pathway are likely to have novel DIC transporters.

Organisms whose genomes include both a carboxysome locus as well as noncarboxysomal RubisCO genes use both CO_2_ and HCO_3_^−^, but unlike DCHB organisms, their use of these forms of DIC is not simultaneous but differentially regulated. Under low CO_2_ conditions, they rely predominantly on HCO_3_^−^ by upregulating carboxysome expression and repressing cytoplasmic RubisCO expression; under high CO_2_ conditions, they rely predominantly on CO_2_ by upregulating cytoplasmic RubisCO expression and repressing carboxysome expression (45, 86, 87). Since they alternate between carboxysomal and noncarboxysomal CBB use, their complement of DIC toolkit genes resembles a combination of both (Fig. 7), with high numbers of DIC transporter genes (similar to organisms that solely encode carboxysomal RubisCO), high numbers of CA genes (similar to both carboxysomal and noncarboxysomal CBB use), and an abundance of eCA genes.

## FURTHER QUESTIONS

The analysis of DIC toolkit components encoded in the genomes of a variety of autotrophic organisms has raised some points of interest for autotrophs in general, as well as points specific to each pathway. One important unknown is the identity and prevalence of yet-to-be-described DIC transporters and CA. The latest additions to the lists of known DIC toolkit components and autotrophic pathways have been relatively recent [newest DIC transporter: 2017 (70); newest CA: 2019 (65); and newest autotrophic DIC fixation pathway: 2020 (39)], suggesting that there is much that remains to be uncovered. Undersampling issues are also apparent: comparatively few members of *Archaea* have been sequenced and studied, only two HP autotrophs have been completely sequenced, and organisms thriving at pH extremes and low temperatures are undersequenced (Fig. 3 to 5). Additionally, the interesting possibilities raised by genome data should be confirmed by measurements of gene expression and function under different growth conditions.

The presence of genes encoding both DIC transporters and cCA in organisms using carboxysomal and non-carboxysomal CBB, rTCA, and HPHB is also curious, given that their high-level coexpression in other organisms provides no growth advantage (125) or causes loss of growth under low CO_2_ conditions (82). Differential expression and modulated expression (116) to minimize leakage are possible, as is a novel form of spatial segregation analogous to transporters and carboxysomes in organisms with CCMs.

The DIC toolkit is especially open for study among organisms using non-CBB pathways for DIC fixation, and the presence of toolkit genes in these organisms raises the possibility of studies of their function and expression. Are the rTCA organisms indeed taxonomically bimodal with respect to their adaptation to growth under low CO_2_ conditions? Does the presence of DIC toolkit genes in rTCA organisms beyond phylum *Chlorobiota* enable them to grow better under low CO_2_ conditions? Are there parallels in DIC transporter and CA expression with CBB organisms? Are WL organisms specifically adapted to high CO_2_ conditions, or do some of them have yet-to-be-described transporter activities [e.g., acetate:HCO_3_^−^ antiporters (94)] that could facilitate growth under low CO_2_ conditions? Given that non-CBB autotrophs include many thermophiles and hyperthermophiles, they provide an opportunity to study the degree to which high temperatures influence the activity and necessity of DIC toolkit capabilities. The addition of more finished genomes from psychrophilic autotrophic organisms could extend these inferences as well (currently, only six are available).

The results of the *in silico* analyses presented here, as well as experimental studies of organisms with CCMs, strongly suggest that DIC toolkit genes could boost the performance of engineered autotrophic organisms in industrial contexts. If these engineered organisms are to be cultivated with air as the source of CO_2_, DIC toolkit genes may be required for growth, as they are in *Cyanobacteria* and *Proteobacteria* with CCMs (70, 80, 128). The prevalence of DIC toolkit genes in autotrophic *Archaea* and *Bacteria* from habitats ranging from pH 1 to pH 11 (Fig. 3 to 5) using all six autotrophic pathways (Fig. 7 and 8) suggests that these genes provide a selective advantage to the organisms that carry them, which may translate into enhanced biomass in an industrial context. Given that organisms with disrupted DIC toolkit genes can be rescued when provided with extremely high CO_2_ concentrations [1%–5% headspace CO_2_, vol/vol (70, 80, 128)], it is possible that organisms in industrial environments with high CO_2_ concentrations will not require DIC toolkit genes. However, many organisms that have been isolated from high CO_2_ environments, as detailed above, have an elaborate collection of DIC toolkit genes, suggesting their utility even in these environments. The technologies for engineering microorganisms have only been available for 50 years (129). Given the fact that microorganisms have been evolving for 3.4–4.2 billion years (130–132), it seems that our attempts to engineer them are best informed by learning from existing organisms from multiple phyla and domains.

## SUPPLEMENTAL MATERIAL

Supplemental figures, tables, and details aem.01557-23-s0001.docx Supplemental material includes Tables S1-S3, Fig. S1, and details about gathering genomes, verifying predicted functions of genes, and calculating demand for CO2 and bicarbonate.

ASM does not own the copyrights to Supplemental Material that may be linked to, or accessed through, an article. The authors have granted ASM a non-exclusive, world-wide license to publish the Supplemental Material files. Please contact the corresponding author directly for reuse.
