# Supplementary material for: Widespread dissolved inorganic carbon-modifying toolkits in genomes of autotrophic Bacteria and Archaea and how they are likely to bridge supply from the environment to demand by autotrophic pathways
Source: Appl Environ Microbiol. 2024 Feb 1;90(2):e01557-23. doi: 10.1128/aem.01557-23 (PMC10880623; doi:10.1128/aem.01557-23)
Supplement: Supplemental figures, tables, and details — Supplemental material includes Tables S1-S3, Fig. S1, and details about gathering genomes, verifying predicted functions of genes, and calculating demand for CO2 and bicarbonate. [file aem.01557-23-s0001.docx]

**SUPPLEMENTAL MATERIAL**

**Table S1. Habitats and physiology of autotrophic organisms chosen for this study**

| **IMG taxon_oid** | **D** | **Genome Name / Sample Name** | **pathway** | **optimal pH^a^** | **Optimal temperature^b^** | **isolation habitat** | **source for pH, isolation, physiology**^c^ |
| --- | --- | --- | --- | --- | --- | --- | --- |
| 650716001 | A | Acidianus hospitalis W1 | HPHB |  | Thermophile | terrestrial acidic hot spring | (1) |
| 2740892001 | A | Acidianus manzaensis YN-25 | HPHB | 1.35 | Thermophile | hot fumarole | (2) |
| 2751185721 | B | Acidihalobacter ferrooxydans V8 | cbb csomes, FII | 1.80 | Mesophile | terrestrial acidic hot spring | (3) |
| 2718218021 | B | Acidihalobacter prosperus V6 | cbb csomes, FII | 2.75 | Mesophile | shallow marine hot spring | (4) |
| 644736322 | B | Acidimicrobium ferrooxidans ICP, DSM 10331 | cbb csomes | 2.00 | Thermophile | terrestrial acidic hot spring | (5) |
| 2811995294 | B | Acidithiobacillus caldus ATCC 51756 | cbb csomes, FII | 2.25 | Mesophile | coal spoils | (6) |
| 2510436001 | B | Acidithiobacillus ferrivorans SS3 | cbb csomes, FI and FII | 2.50 | Mesophile | copper mine spoil drainage | (7) |
| 643348501 | B | Acidithiobacillus ferrooxidans ATCC 23270 | cbb csomes, FI and FII | 2.50 | Mesophile | acid mine drainage | (6) |
| 651053055 | B | Afipia carboxidovorans OM4 | cbb FI only | *7.00* | Mesophile | waste water | (8) |
| 637000005 | B | Alkalilimnicola ehrlichii MLHE-1 | cbb FI only | 9.30 | Mesophile | alkaline hypersaline soda lake | (9) |
| 646564502 | B | Allochromatium vinosum DSM 180 | cbb csomes, FI | 7.15 | Mesophile | ditch water | (10) |
| 637000010 | B | Aquifex aeolicus VF5 | rTCA | 6.80 | Hyperthermophile | shallow marine hot spring | (11) |
| 638154502 | A | Archaeoglobus fulgidus VC-16, DSM 4304 | WL | 6.50 | Hyperthermophile | shallow marine hot spring | (12) |
| 2522125074 | A | Archaeoglobus sulfaticallidus PM70-1, DSM 19444 | WL | 7.00 | Thermophile | borehole | (12) |
| 2751185563 | B | Bathymodiolus septemdierum endosymbiont Myojin knoll A1 | cbb FII only |  | Mesophile | intracellular | (13) |
| 2974486711 | B | Bathymodiolus thermophilus thioautotrophic gill symbiont EPR9N | cbb FI only |  | Mesophile | intracellular | (14) |
| 2788500402 | B | Beggiatoa leptomitoformis D-401 | cbb FI only | 7.65 | Mesophile | freshwater spring contaminated with wastewater | (15) |
| 637000038 | B | Bradyrhizobium diazoefficens USDA 110 | cbb FI only | 6.80 | Mesophile | soil, rhizosphere | (16), (17) |
| 2964078231 | B | Caldichromatium japonicum No.7 | cbb csomes, FI | 7.20 | Thermophile | terrestrial hot spring | (18) |
| 2788500263 | A | Candidatus Nitrosocaldus islandicus | HPHB |  | Thermophile | terrestrial hot spring | (19) |
| 2757320762 | A | Candidatus Nitrosomarinus catalina SPOT01 | HPHB |  | Mesophile | subsurface Pacific waters | (20) |
| 2630968793 | A | Candidatus Nitrosopelagicus brevis CN25 | HPHB |  | Mesophile | open ocean | (21) |
| 2518645532 | A | Candidatus Nitrosopumilus koreensis AR1 | HPHB | *8.20* | Mesophile | marine sediment | (22) |
| 2518645576 | A | Candidatus Nitrosopumilus sediminis AR2 | HPHB | *8.20* | Psychrophile | arctic marine sediment | (23) |
| 2585427666 | A | Candidatus Nitrososphaera evergladensis SR1 | HPHB |  | Mesophile | everglades soil | (24) |
| 2510065023 | A | Candidatus Nitrososphaera gargensis Ga9-2 | HPHB | *7.80* | Thermophile | terrestrial hot spring | (25) |
| 2757320681 | A | Candidatus Nitrosotalea devanaterra NDEV1 | HPHB | 4.50 | Mesophile | acidic agricultural soil | (26) |
| 2775506851 | A | Candidatus Nitrosotalea okcheonensis CS NCS1 | HPHB | 7.25 | Mesophile | deep oligotrophic soil horizon | (27) |
| 2630968650 | A | Candidatus Nitrosotenuis cloacae SAT1 | HPHB | 6.25 | Mesophile | activated sludge | (28) |
| 649633030 | B | Candidatus Nitrospira defluvii | rTCA |  | Mesophile | sewage | (29), (30) |
| 2684623072 | B | Candidatus Nitrospira inopinata ENR4 | rTCA |  | Thermophile | pipe of a deep oil exploration well | (31) |
| 2909959871 | B | Candidatus Ruthia endofausta JDF_Ridge | cbb FII only |  | Mesophile | intracellular | (32) |
| 639633019 | B | Candidatus Ruthia magnifica Cm | cbb FII only |  | Mesophile | intracellular | (33) |
| 8003201789 | B | Candidatus Sulfurimonas baltica GD2 | rTCA | 7.25 | Psychrophile | pelagic redoxcline | (34) |
| 2996021010 | B | Candidatus Sulfurimonas marisnigri SoZ1 | rTCA | 7.75 | Mesophile | pelagic redoxcline | (34) |
| 2751185675 | B | Candidatus Tenderia electrophaga NRL1 | cbb csomes, FI |  | Mesophile | biocathode | (35) |
| 2773857920 | B | Candidatus Thiodictyon syntrophicum Cad16 | cbb csomes, FII | 7.15 | Mesophile | chemocline of a lake | (36) |
| 2645727956 | B | Candidatus Thioglobus autotrophicus EF1 | cbb FI only |  | Mesophile | redox gradient of marine inlet | (37) |
| 2816332301 | B | Candidatus Thioglobus singularis GG2 | cbb FI only |  | Mesophile | marine surface water | (38) |
| 640427107 | B | Candidatus Vesicomyosocius okutanii HA | cbb FII only |  | Mesophile | intracellular | (39) |
| 642555120 | B | Chlorobaculum parvum DSM 263 | rTCA | *7.00* | Mesophile | waste water | (40) |
| 637000073 | B | Chlorobaculum tepidum TLS | rTCA | 6.90 | Thermophile | acidic freshwater hot spring | (41) |
| 637000072 | B | Chlorobium chlorochromatii CaD3 | rTCA | 7.15 | Mesophile | eutrophic lake | (42) |
| 642555121 | B | Chlorobium limicola DSM 245 | rTCA | 6.80 | Mesophile | terrestrial hot spring | (40) |
| 642555122 | B | Chlorobium phaeobacteroides BS1 | rTCA | 6.90 | Mesophile | saline intertidal flat | (43) |
| 640427130 | B | Chlorobium phaeovibrioides DSM 265 | rTCA | 6.90 | Mesophile | saline intertidal flat | (43) |
| 643348527 | B | Chloroflexus aggregans DSM 9485 | HP bicycle | 8 | Thermophile | terrestrial hot spring | (44) |
| 641228485 | B | Chloroflexus aurantiacus J-10-fl | HP bicycle | 8.00 | Thermophile | terrestrial hot spring | (45) |
| 642555123 | B | Chloroherpeton thalassium ATCC 35110 | rTCA | 6.90 | Mesophile | marine sediment | (46) |
| 2609459698 | B | Clostridium aceticum, DSM 1496 | WL | 8.30 | Mesophile | mud | (47) |
| 640427136 | B | Cupriavidus necator H16 | cbb FI only | 7.5 | Mesophile | soil | (48) |
| 2561511172 | B | Desulfocapsa sulfexigens DSM 10523 | WL | 7.00 | Mesophile | marine sediment | (49) |
| 2515154209 | B | Desulfofundulus kuznetsovii DSM 6115 | rTCA | *6.90* | Mesophile | deep subsurface water | (50) |
| 643692021 | B | Desulforapulum autotrophicum HRM2 | WL | 6.70 | Mesophile | freshwater mud | (51) |
| 2523231053 | B | Desulfotignum phosphitoxidans FiPS-3, DSM 13687 | WL | 7.20 | Mesophile | marine sediment | (52) |
| 649633039 | B | Desulfurobacterium thermolithotrophum BSA, DSM 11699 | rTCA | 6.00 | Thermophile | deep sea hydrothermal vent | (53) |
| 2834899978 | B | Ferriphaselus amnicola OYT1 | cbb FII only | 6.30 | Mesophile | groundwater seep | (54) |
| 646564534 | A | Ferroglobus placidus AEDII12DO, DSM 10642 | WL | 7.00 | Hyperthermophile | shallow marine hot spring | (55) |
| 2740892000 | A | Ferroplasma acidiphilum Y | WL | 1.70 | Mesophile | bioleaching plant | (56) |
| 648028028 | B | Gallionella capsiferriformans ES-2 | cbb FII only |  | Mesophile | groundwater | (57) |
| 2634166507 | A | Geoglobus acetivorans SBH6 | WL | 6.80 | Hyperthermophile | deep sea hydrothermal vent | (58) |
| 2808606696 | A | Geoglobus ahangari 234 | WL | 7.00 | Hyperthermophile | deep sea hydrothermal vent | (59) |
| 2571042483 | B | Halorhodospira halochloris A | cbb csomes, FI | 8.60 | Mesophile | extremely saline and alkaline lakes | (10) |
| 639633026 | B | Halorhodospira halophila SL1 | cbb FI only | 8.75 | Mesophile | salt lake mud | (10) |
| 646311935 | B | Halothiobacillus neapolitanus c2, ATCC 23641 | cbb csomes, FII | 6.70 | Mesophile | seawater | (60) |
| 650377953 | B | Hydrogenobacter thermophilus TK-6, DSM 6534 | rTCA | *7.00* | Thermophile | terrestrial hot spring | (61) |
| 2506210035 | B | Hydrogenobaculum sp. 3684 | rTCA |  | Thermophile | terrestrial hot spring | (62) |
| 642555132 | B | Hydrogenobaculum sp. Y04AAS1 | rTCA |  | Thermophile | terresrial hot spring | (63) |
| 2841100635 | B | Hydrogenophaga pseudoflava DSM 1084 | cbb FI only | *7.00* | Mesophile | freshwater | (64) |
| 637000325 | B | Hydrogenovibrio crunogenus XCL-2 | cbb csomes, FI and FII | 7.75 | Mesophile | deep sea hydrothermal vent | (65) |
| 2836772259 | B | Hydrogenovibrio thermophilus JR-2 | cbb csomes, FI and FII | 6.00 | Mesophile | soda lake | (66) |
| 640753029 | A | Ignicoccus hospitalis KIN4/I, DSM 18386 | DCHB | 5.50 | Hyperthermophile | deap sea hydrothermal vent | (67) |
| 2775506848 | B | Kyrpidia spormannii EA-1 | cbb FI only | 5.50 | Thermophile | shallow marine hot spring | (68) |
| 646564511 | B | Kyrpidia tusciae T2, DSM 2912 | cbb FI only | 4.50 | Thermophile | solfatara | (69) |
| 2775507268 | B | Lebetimonas sp. JH292 | rTCA |  | Thermophile | deep sea hydrothermal vent | (70) |
| 2576861452 | B | Leptospirillum ferriphilum YSK | rTCA | 1.6 | Mesophile | acid mine drainage | (71) |
| 2540341086 | B | Leptospirillum ferrooxidans C2-3 | rTCA | 2.75 | Mesophile | volcanic ash | (72) |
| 639633036 | B | Magnetocccus marinus MC-1 | rTCA | 7.0 | Mesophile | Oxic/anoxic interface of an estuary | (73) |
| 2510065050 | B | Marichromatium purpuratum 984 | cbb csomes, FI | 7.00 | Mesophile | saline lake | (74) |
| 2775506950 | B | Mariprofundus aestuarium CP-5 | cbb FII only | 7.05 | Mesophile | Chesapeake bay oxic–anoxic transition zone | (75) |
| 2775506949 | B | Mariprofundus ferrinatatus CP-8 | cbb FII only | 7.05 | Mesophile | Chesapeake bay oxic–anoxic transition zone | (75) |
| 650716051 | A | Metallosphaera cuprina Ar-4 | HPHB | 3.50 | Thermophile | terrestrial hot spring | (76) |
| 640427120 | A | Metallosphaera sedula DSM 5348 | HPHB | 2.75 | Thermophile | solfataras | (77) |
| 2630968343 | A | Methanobacterium formicicum BRM9 | WL | *6.90* | Mesophile | cow rumen | (78) |
| 650716052 | A | Methanobacterium lacus AL-21 | WL | 6.20 | Mesophile | peatlands | (79) |
| 650716053 | A | Methanobacterium paludis SWAN-1 | WL | 5.55 | Mesophile | peatlands | (79) |
| 644736385 | A | Methanocaldococcus fervens AG86 | WL | 6.50 | Thermophile | deep sea hydrothermal vent | (80) |
| 646564547 | A | Methanocaldococcus infernus ME | WL | 6.50 | Thermophile | deep sea hydrothermal vent | (81) |
| 638154505 | A | Methanocaldococcus jannaschii DSM 2661 | WL | 6.00 | Hyperthermophile | deep sea hydrothermal vent | (82) |
| 646311944 | A | Methanocaldococcus vulcanius M7, DSM 12094 | WL | 6.50 | Thermophile | deep sea hydrothermal vent | (83) |
| 2505679073 | A | Methanocella arvoryzae MRE50 | WL | 7.00 | Mesophile | soil | (84) |
| 640753034 | A | Methanococcus aeolicus Nankai-3 | WL | *6.90* | Mesophile | deep sea sediment | (85) |
| 640069316 | A | Methanococcus maripaludis C5 | WL | 7.00 | Mesophile | salt-marsh sediment | (86) |
| 648028039 | A | Methanohalobium evestigatum Z-7303, DSM 3721 | WL | *7.40* | Thermophile | saline lagoons | (87) |
| 638154507 | A | Methanopyrus kandleri AV19 | WL | 6.50 | Hyperthermophile | deep sea hydrothermal vent | (88) |
| 640753014 | A | Methanoregula boonei 6A8 | WL | 5.10 | Mesophile | acidic bog | (89) |
| 2634166439 | A | Methanosarcina barkeri CM1 | WL | 7.00 | Mesophile | sewage sludge | (90) |
| 637000164 | A | Methanospirillum hungatei JF-1 | WL | 7.00 | Mesophile | sewage sludge | (91) |
| 648028041 | A | Methanothermobacter marburgensis Marburg DSM 2133 | WL | 7.10 | Thermophile | sewage sludge | (92) |
| 638154510 | A | Methanothermobacter thermautotrophicus Delta H | WL | 7.40 | Thermophile | sewage sludge | (92) |
| 650716055 | A | Methanothermococcus okinawensis IH1 | WL | 6.5 | Thermophile | deep sea hydrothermal vent | (93) |
| 650716056 | A | Methanotorris igneus Kol5, DSM 5666 | WL | 6.70 | Hyperthermophile | deep sea hydrothermal vent | (94) |
| 637000167 | B | Moorella thermoacetica ATCC 39073 | WL | *7.30* | Thermophile | horse feces | (95) |
| 643692029 | B | Nautilia profundicola Am-H | rTCA | 7.00 | Thermophile | deep sea hydrothermal vent | (96) |
| 2836927353 | B | Nautilia sp. PV-1 | rTCA |  | Thermophile | deep sea hydrothermal vent | (97) |
| 649633076 | B | Nitratifractor salsuginis E9I37-1, DSM 16511 | rTCA | 6.40 | Mesophile | deep sea hydrothermal vent | (98) |
| 2989147754 | B | Nitratiruptor sp. EPR5501 | rTCA | 6.6 | Thermophile | deep sea hydrothermal vent | (99) |
| 640753037 | B | Nitratiruptor sp. SB155-2 | rTCA |  | Thermophile | deep sea hydrothermal vent | (98) |
| 3000948971 | B | Nitratiruptor sp. YY08-10 | rTCA |  | Thermophile | deep sea hydrothermal vent | (100) |
| 3000934859 | B | Nitratiruptor sp. YY-09-18 | rTCA |  | Thermophile | deep sea hydrothermal vent | (100) |
| 637000192 | B | Nitrobacter hamburgensis X14 | cbb csomes, FI | 7.75 | Mesophile | soil | (101) |
| 637000193 | B | Nitrobacter winogradskyi Nb-255 | cbb csomes, FI | 7.75 | Mesophile | soil, water, sewage | (102) |
| 646564556 | B | Nitrosococcus halophilus Nc4 | cbb FI only |  | Mesophile | salt lake | (103) |
| 637000194 | B | Nitrosococcus oceani C-107 | cbb FI only | 7.65 | Mesophile | open ocean waters | (104) |
| 648028046 | B | Nitrosococcus watsonii C-113 | cbb FI only | 7.80 | Mesophile | oxygen minimum zones of open ocean | (105) |
| 2657244923 | A | Nitrosocosmicus oleophilus MY3 | HPHB | 6.75 | Mesophile | coal tar-contaminated sediment | (106) |
| 2627854142 | B | Nitrosomonas communis Nm2 | cbb FI only | *7.80* | Mesophile | soil | (107) |
| 637000195 | B | Nitrosomonas europaea ATCC 19718 | cbb FI only | 7.75 | Mesophile | soil | (107) |
| 637000196 | B | Nitrosomonas eutropha C91 | cbb csomes | *7.80* | Mesophile | sewage | (107) |
| 2885017570 | B | Nitrosomonas stercoris KYUHI-S | cbb FI only | 8.00 | Mesophile | composted cattle manure | (108) |
| 2989009520 | B | Nitrosophilus labii HRV44 | rTCA | 6 | Thermophile | deep sea hydrothermal vent | (109) |
| 2627854092 | A | Nitrosopumilus adriaticus NF5 | HPHB | 7.20 | Mesophile | coastal surface water | (110) |
| 641228499 | A | Nitrosopumilus maritimus SCM1 | HPHB | 7.10 | Mesophile | marine | (111) |
| 2988910918 | A | Nitrosopumilus oxyclinae HCE1 | HPHB | 7.30 | Mesophile | coastal surface water | (110) |
| 2627853696 | A | Nitrosopumilus piranensis D3C | HPHB | 7.30 | Mesophile | coastal surface water | (110) |
| 2585427612 | A | Nitrososphaera viennensis EN76 | HPHB | 7.50 | Mesophile | soil | (112) |
| 2642422553 | B | Nitrosospira lacus APG3 | cbb FI only | 7.5 | Mesophile | lake sediment | (113) |
| 637000197 | B | Nitrosospira multiformis ATCC 25196 | cbb FI only | 7.50 | Mesophile | soil | (114) |
| 2757321055 | B | Nitrospira japonica NJ11 | rTCA |  | Mesophile | seawage | (115) |
| 2639762945 | B | Nitrospira moscoviensis NSP M-1 | rTCA | 7.80 | Mesophile | corroded iron pipe | (116) |
| 2883930301 | B | Nitrospira sp. KM1 | rTCA |  | Mesophile | drinking water treatment plant | (117) |
| 649989977 | B | Oscillochloris trichoides DG6 | cbb FI only | 8.25 | Mesophile | terrestrial hot spring | (118) |
| 637000205 | B | Pelodictyon luteolum DSM 273 | rTCA | 6.75 | Mesophile | lake | (119) |
| 642555146 | B | Pelodictyon phaeoclathratiforme BU-1 | rTCA | 6.60 | Mesophile | stratified lake | (119) |
| 643692030 | B | Persephonella marina EX-H1 | rTCA | 6.00 | Thermophile | deep sea hydrothermal vent | (120) |
| 642555149 | B | Prosthecochloris aestuarii SK413, DSM 271 | rTCA | 7.40 | Mesophile | brackish sediment | (119) |
| 2721755577 | B | Prosthecochloris sp. CIB 2401 | rTCA |  |  | hypersaline lake | (121) |
| 2757320899 | B | Prosthecochloris sp. GSB1 | rTCA |  | Mesophile | deep sea hydrothermal vent | (122) |
| 2751185712 | B | Prosthecochloris sp. HL-GSB HL-130-GSB | rTCA |  | Mesophile | saline lake | (123) |
| 2775506867 | B | Pseudodesulfovibrio profundus 500-1 | cbb FII only | 6.75 | Mesophile | deep sea serpentinized peridotite | (124) |
| 2856823606 | B | Pseudonocardia autotrophica NBRC 12743 | cbb FI only | *7.30* | Mesophile | phosphate buffer solution from a lab | (125) |
| 651053061 | B | Pseudonocardia dioxanivorans CB1190 | cbb FI only | *7.20* | Mesophile | dioxane-contaminated industrial sludge | (126) |
| 2636415851 | B | Pseudorhizobium banfieldiae NT-26 | cbb FI only | 7.5 | Mesophile | sub-surface goldmine | (127) |
| 638154513 | A | Pyrobaculum aerophilum IM2 | DCHB | 7.00 | Hyperthermophile | shallow marine hot spring | (128) |
| 639633053 | A | Pyrobaculum islandicum DSM 4184 | DCHB | 6.00 | Hyperthermophile | neutral and alkaline sulfotaras | (129), (130) |
| 641522657 | A | Pyrobaculum neutrophilum V24Sta | DCHB | 6.50 | Hyperthermophile | terrestrial hot spring | (130), (131) |
| 2505679005 | A | Pyrolobus fumarii 1A, DSM 11204 | DCHB | 5.50 | Hyperthermophile | deep sea hydrothermal vent | (132) |
| 646564563 | B | Rhodobacter capsulatus SB1003 | cbb FI only | 7.00 | Mesophile | fresh water | (133) |
| 2751185768 | B | Rhodoferax antarcticus DSM 24876 | cbb FI only | *6.80* | Psychrophile | antarctic marine sediment | (134) |
| 639279312 | B | Rhodopseudomonas palustris BisA53 | cbb FI only | *7.10* | Mesophile | fresh water and mud | (135) |
| 643348571 | B | Rhodospirillum centenum SW | cbb FI only | *6.80* | Mesophile | terrestrial hot spring | (136) |
| 2511231162 | B | Rhodospirillum rubrum F11 | cbb FII only | 6.90 | Mesophile | fresh water | (133) |
| 2687453592 | B | Rhodovulum sulfidophilum DSM 1374 | cbb FI only | 7.25 | Mesophile | marine mud | (137) |
| 2513237199 | B | Rubrivivax gelatinosus IL144 | cbb FI only | 7 | Mesophile | mud | (138) |
| 2654588029 | B | Sedimenticola thiotaurini SIP-G1 | cbb FII only | *7.60* | Mesophile | salt marsh sediment | (139) |
| 2630968402 | B | Serpentinimonas maccroryi B1 | cbb csomes | 11.00 | Mesophile | terrestrial serpentinizing springs | (140) |
| 2636415698 | B | Serpentinomonas raichei A1 | cbb csomes | 11.00 | Mesophile | terrestrial serpentinizing springs | (140) |
| 646564569 | B | Sideroxydans lithotrophicus ES-1 | cbb FI only | 6.25 | Mesophile | deep sea hydrothermal vent | (141) |
| 637000269 | B | Sinorhizobium meliloti 1021 | cbb FI only | *7.00* | Mesophile | soil, rhizosphere | (142) |
| 648028054 | B | Starkeya novella DSM 506 | cbb FI only | 7.00 | Mesophile | soil | (143) |
| 2506520015 | B | Sulfobacillus acidophilus NAL, DSM 10332 | cbb FI only | 2.00 | Thermophile | coal spoils | (144) |
| 646311959 | A | Sulfolobus islandicus L.D.8.5 | HPHB | 3.25 | Hyperthermophile | terrestrial hot spring | (145) |
| 2728369534 | A | Sulfolobus sp. A20 | HPHB | 3.25 | Thermophile | terrestrial acidic hot spring | (146) |
| 2775506817 | B | Sulfuricaulis limicola HA5 | cbb FI only | *7.10* | Mesophile | lake sediment | (147) |
| 2531839478 | B | Sulfuricella denitrificans skB26 | cbb FI only | 7.50 | Mesophile | lake sediment | (148) |
| 649633097 | B | Sulfuricurvum kujiense YK-1, DSM 16994 | rTCA | 7.00 | Mesophile | underground crude-oil storage cavity | (149) |
| 2884026358 | B | Sulfuriferula nivalis SGTM | cbb csomes, FI and FII | 6.60 | Psychrophile | snow | (150) |
| 2884029866 | B | Sulfuriferula plumbiphila Gro7 | cbb csomes, FI | 6.70 | Mesophile | uranium mine | (150) and (151) |
| 2837265499 | B | Sulfuriflexus mobilis aks1 | cbb csomes, FI | 7.50 | Mesophile | brakish lake sediment | (152) |
| 2775506818 | B | Sulfurifustis variabilis skN76 | cbb FI only | 7.50 | Mesophile | lake sediment | (148) |
| 643692050 | B | Sulfurihydrogenibium azorense Az-Fu1 | rTCA | 6.00 | Thermophile | terrestrial hot spring | (153) |
| 642555165 | B | Sulfurihydrogenibium yellowstonense YO3AOP1 | rTCA | 7.50 | Thermophile | terrestrial hot spring | (154) |
| 2898025466 | B | Sulfurimicrobium lacus skT11 | cbb FI only | 6.70 | Mesophile | lake water | (155) |
| 3003276765 | B | Sulfurimonas aquatica H1576 | rTCA | 6.7 | Psychrophile | brackish lake water | (156) |
| 648028058 | B | Sulfurimonas autotrophica OK10, DSM 16294 | rTCA | 6.50 | Mesophile | deep sea hydrothermal vent | (157) |
| 637000326 | B | Sulfurimonas denitrificans DSM 1251 | rTCA | 7.00 | Mesophile | coastal marine sediments | (158) |
| 2811995045 | B | Sulfurimonas gotlandica GD1 | rTCA | 7.30 | Mesophile | pelagic redoxcline | (159) |
| 3003274224 | B | Sulfurimonas hydrogeniphila NW10 | rTCA | 6.25 | Mesophile | deep sea hydrothermal vent | (160) |
| 2887920885 | B | Sulfurimonas lithotrophica GYSZ_1 | rTCA | 6.50 | Mesophile | marine sediment | (161) |
| 3003279535 | B | Sulfurimonas marina B2 | rTCA | 7 | Mesophile | deep sea sediment | (162) |
| 2996014676 | B | Sulfurimonas paralvinellae GO25 | rTCA | 6.1 | Mesophile | deep sea hydrothermal vent | (158) |
| 2911865633 | B | Sulfurimonas sediminis S2-6 | rTCA | 7.00 | Mesophile | deep sea hydrothermal vent | (163) |
| 2887923346 | B | Sulfurimonas sp. CVO | rTCA |  | Psychrophile | brine at an oil field | (164) |
| 638154519 | A | Sulfurisphaera tokodaii 7 | HPHB | 3.75 | Hyperthermophile | acidic terrestrial hot spring | (165) |
| 2836985240 | B | Sulfuritortus calidifontis J1A | cbb csomes?, FI?, FII | 7.05 | Mesophile | terrestrial hot spring | (166) |
| 2837194640 | B | Sulfurivermis fontis JG42 | cbb FI only | 7.55 | Mesophile | terrestrial hot spring | (167) |
| 2648501445 | B | Sulfurovum lithotrophicum ATCC BAA-797 | rTCA | 6.75 | Mesophile | deep sea hydrothermal vent | (168) |
| 2989423809 | B | Sulfurovum indicum ST-419 | rTCA | 6 | Mesophile | deep sea hydrothermal vent | (169) |
| 640753056 | B | Sulfurovum sp. NBC37-1 | rTCA |  | Thermophile | deep sea hydrothermal vent | (170) |
| 646564582 | B | Thermocrinis albus HI 11/12, DSM 14484 | rTCA | *7.00* | Hyperthermophile | terrestrial hot spring | (171) |
| 2512875013 | B | Thermocrinis ruber DSM 23557 | rTCA | 7.80 | Hyperthermophile | terrestrial hot spring | (172) |
| 2505119042 | B | Thermodesulfatator indicus CIR29812, DSM 15286 | WL | 6.25 | Thermophile | deep sea hydrothermal vent | (173) |
| 2511231093 | A | Thermoproteus tenax Kra1 | DCHB | 5.00 | Thermophile | acidic terrestrial hot spring | (174) |
| 649633104 | B | Thermovibrio ammonificans HB-1 | rTCA | 5.50 | Thermophile | deep sea hydrothermal vent | (175) |
| 2521172692 | B | Thioalkalivibrio nitratireducens DSM 14787 | cbb csomes | 9.75 | Mesophile | soda lake | (176) |
| 2506520043 | B | Thioalkalivibrio paradoxus ARh 1 | cbb csomes | 10.20 | Mesophile | soda lake | (177) |
| 643348585 | B | Thioalkalivibrio sulfidophilus HL-EbGR7 | cbb csomes | 10.00 | Mesophile | soda lake | (178) |
| 2630968613 | B | Thioalkalivibrio versutus D301 | cbb csomes | 10.10 | Mesophile | soda lake | (179) |
| 637000324 | B | Thiobacillus denitrificans ATCC 25259 | cbb csomes?, FI?, FII | 7.10 | Mesophile | soil and sediments | (180) |
| 2508501051 | B | Thiocystis violascens 611, DSM 198 | cbb FI only | 7.15 | Mesophile | stagnant freshwater, sewage, estuaries, salt marshes, and sulfur springs | (10) |
| 2506783059 | B | Thioflavicoccus mobilis 8321 | cbb csomes, FI and FII | 7.30 | Mesophile | salt marsh sediments | (181) |
| 2775506810 | B | Thiohalobacter thiocyanaticus FOKN1 | cbb csomes, FI | 7.40 | Mesophile | hypersaline lake | (182) |
| 2989024789 | B | Thiolapillus brandeum Hiromi1 | cbb FII only | 6.50 | Mesophile | deep sea hydrothermal vent | (183) |
| 2839896438 | B | Thiomicrorhabdus aquaedulcis HaS4 | cbb csomes, FII | 7.00 | Mesophile | lake water | (184) and (185) |
| 2836887846 | B | Thiomicrorhabdus indica 13-15A | cbb csomes, FII | 7.00 | Mesophile | hydrothermal vent | (186) |
| 2506783063 | B | Thiomicrospira aerophila AL3 | cbb csomes | 9.5 | Mesophile | soda lake | (179) |
| 2505679009 | B | Thiomicrospira cyclica ALM1 | cbb csomes | 9.50 | Mesophile | alkaline hypersaline lake | (187) |
| 651053077 | B | Thiomonas arsenitoxydans 3As | cbb csomes, FII | 5.00 | Mesophile | acid mine water | (188) |
| 2772190779 | B | Thiomonas intermedia ATCC 15466 | cbb csomes, FII |  | Mesophile | freshwater mud | (189) |
| 2619619276 | B | Thioploca ingrica Lake Okotanpe | cbb FI only |  | Mesophile | freshwater sediment | (190) and (191) |
| 2509276036 | B | Thiorhodospira sibirica A12, ATCC 700588 | cbb FI only | 9.00 | Mesophile | soda lake | (192) |
| 2884036832 | B | Thiosulfatimonas sediminis aks77 | cbb csomes | 7.45 | Mesophile | brakish lake sediment | (193) |
| 2884039451 | B | Thiosulfativibrio zosterae AkT22 | cbb csomes, FI and FII | 6.95 | Mesophile | brakish lake | (193) |
| 650716102 | B | Treponema primitia ZAS-2 | WL | 7.20 | Mesophile | termite hindguts | (194) |
| 640753059 | B | Xanthobacter autotrophicus Py2 | cbb FI only | *6.80* | Mesophile | ditch water | (195) |

^a^growth medium pH from https://bacdive.dsmz.de/ in italics if optimum not available

^b^psychrophile: -20 to 20°C; mesophile: 20-45°C; thermophile: 45 – 80°C; hyperthermophile: 80-122°C

^c^References were converted from doi to Endnote citations using a script located at [https://github.com/scooterboi85/RIS-converter](https://nam04.safelinks.protection.outlook.com/?url=https%3A%2F%2Fgithub.com%2Fscooterboi85%2FRIS-converter&data=05%7C01%7Ckmscott%40usf.edu%7C6bad106eaf61447356e308dbab33e0c1%7C741bf7dee2e546df8d6782607df9deaa%7C0%7C0%7C638292011597274426%7CUnknown%7CTWFpbGZsb3d8eyJWIjoiMC4wLjAwMDAiLCJQIjoiV2luMzIiLCJBTiI6Ik1haWwiLCJXVCI6Mn0%3D%7C3000%7C%7C%7C&sdata=bMd5gWNTrCiuw92DJbx9M1Avb24%2Btl%2FjkeTh1kz0ZVw%3D&reserved=0)

**Gathering finished genomes from autotrophic members of *Bacteria* and *Archaea* from IMG**

Genomes from organisms using the CBB cycle were selected from IMG by gathering those encoding carboxysomal and noncarboxysomal form I RubisCO (*cbbLS*), as well as form II RubisCO (*cbbM*). An initial long list of potential CBB genomes was created by gathering all finished bacterial genomes (except those belonging to “*Cyanobacteria”*) carrying genes belonging to Pfam00016 (RubisCO large subunit), and was trimmed to include only one representative of each species. Members of this list whose genomes included genes belonging to Pfam00101 (form I RubisCO small subunit) adjacent on the genome to those encoding RubisCO large subunits were placed on a list of FI CBB genomes. This list was further subdivided into cytoplasmic and carboxysomal FI CBB genomes by removing the genomes that included genes belonging to Pfam12288 (CsoS2) adjacent to *cbbL*  and *cbbS,* and adding them to a new list of carboxysomal CBB genomes. The list of carboxysomal CBB genomes was further split into a list of those only encoding carboxysomal RubisCO, and a list of those encoding both a carboxysomal and cytoplasmic RubisCO. A list of FII CBB genomes was created by examining the genes from Pfam00016 that lack a *cbbS* gene nearby. In order to exclude genes encoding RubisCO-like proteins, the amino acid sequences predicted from the genes were aligned with biochemically characterized form I and form II RubisCO and RubisCO-like proteins and a neighbor-joining analysis was undertaken to find the sequences clustering with the form II RubisCOs. These potential form II RubisCO sequences were examined for the presence of residues necessary for carboxylase activity (corresponding to K 215, D217, E218 from form II RubisCO from *R. rubrum;* PDB 5RUB; (196)). Those with residues indicating a form II Rubisco that could catalyze CO_2_ fixation were added to a list of form II CBB genomes. Any on this list with a carboxysome operon were added to the list of organisms encoding both a carboxysomal and cytoplasmic RubisCO. The lists of organisms encoding only cytoplasmic form I RubisCO and/or form II RubisCO were combined as a list of all CBB organisms lacking carboxysomes. At this point, three lists of genomes had been generated: CBB (no carboxysomes), Csomes + nonCS (form I, form II, or both), and Csomes. The presence of genes encoding the other enzyme unique to the CBB (phosphoribulokinase, E.C. 2.7.1.19; Pfam00485) was verified in all of these genomes, and all have a documented ability to grow autotrophically (Table S1).

It was not possible to use a process parallel to gathering CBB organisms for rTCA, as there is not a Pfam specific to ATP citrate lyase. Instead, rTCA genomes were selected from IMG by creating an initial list of finished genomes carrying genes falling within KEGG Enzyme EC 2.3.3.8 (ATP citrate lyase, α and β subunits). Since citrate-CoA ligase (EC 6.2.1.18) and citryl-CoA lyase (EC 4.1.3.34) can also act together to catalyze the first step of the rTCA (197), genomes were gathered using KEGG Enzyme EC 6.2.1.18. These genomes were checked for the presence of genes encoding citrate-CoA ligase by using Pfams 02629 and 00549 (α subunit), and 00549 and 08442 (β subunit). The presence of citryl-CoA lyase was verified using Pfam00285. *Magnetococcus marinus* and *Leptospirillum* spp. were added to the list of potential rTCA organisms based on the literature about these organisms and the observation that they have genes encoding an alternative ATP citrate lyase (71-73, 198). Once a list of genomes with a mechanism for cleaving citrate was created, it was shortened by trimming out genomes that did not include genes encoding the 2-oxoacid oxidoreductases necessary for carboxylating acetyl-CoA (pyruvate synthase; EC 1.2.7.1) or succinyl-CoA (2-oxoglutarate synthase; EC 1.2.7.3; α subunit: Pfam01855 pyruvate flavodoxin/ferrodoxin oxidoreductase, thiamine diP-bdg; Pfam17147 Pyruvate:ferredoxin oxidoreductase core domain II; β subunit: Pfam02775 Thiamine pyrophosphate enzyme, C-terminal TPP binding domain). The ability of the organisms on this list of rTCA genomes to grow autotrophically was verified by examining the literature (Table S1).

To create a list of genomes from organisms capable of using the WL pathway to grow autotrophically, IMG was searched for genomes carrying genes encoding the two CO_2_-fixing steps necessary to synthesize acetyl-CoA (KEGG Enzyme EC 1.2.7.4, CO dehydrogenase; EC 2.3.1.169 CO-methylating acetyl-CoA synthase). These genomes were examined for the presence of genes with the potential to encode pyruvate synthase and 2-oxoglutarate synthase as described above for rTCA genomes. As the WL pathway can be used either as an autotrophic CO_2_-fixing pathway or for fermentation, this list of genomes was shortened by selecting those listed in IMG as both as autotrophs or lithotrophs and methanogens or acetogens. Their ability to grow autotrophically was verified by checking the literature (Table S1).

Creating a list of organisms capable of growing autotrophically via the HP bicycle was simplified by the fact that thus far, only members of phylum *Chloroflexi* have been verified to use this pathway in this manner (199). Accordingly, the literature was examined for members of this phylum confirmed to grow autotrophically by this pathway (Table S1).

Creating lists of HPHB and DCHB genomes began with a single list of finished genomes from domain *Archaea* encoding 4-hydroxybutyryl-CoA dehydratase (KEGG Enzyme EC 4.2.1.120), as this enzyme is shared in both the HPHB and DCHB cycles (200). Genomes on this list that included genes encoding acetyl-CoA carboxylase (Enzyme EC 6.4.1.2) were used to create the HPHB genomes list. The remaining genomes were examined for the presence of genes encoding pyruvate synthase (as described above for rTCA genomes); those including these genes were incorporated into the list of DCHB genomes. The ability of all organisms on the lists to grow autotrophically was verified by examining the literature (Table S1). It is noted in the text that the pathway used by *Pyrobaculum* spp. is in dispute.

**Verifying predicted functions for DIC transporter and carbonic anhydrase genes**

Genes were gathered from IMG from the target genomes based on their membership in Pfams including DIC transporters and carbonic anhydrase enzymes (Table S2). For potential transporters, sequences were examined for predicted transmembrane helices via TMHMM2.0 (201) (DAC-M, SulP, SbtA), and genome colocation (Table S2). Genes encoding likely carbonic anhydrase enzymes were aligned in MEGA11 via MUSCLE (202, 203) and examined for the presence of active site residues (Table S2). Sequences lacking the evidence for function listed in Table S2 were discarded.

**Table S2. Evidence for the function of gene products of homologs of DIC transporter and carbonic anhydrase genes**

| **Gene type** | **Evidence for function** | **Reference** |
| --- | --- | --- |
| **DIC transporters** |  |  |
| DAC-C (Pfam11070) | Colocation with a DAC-M gene in an apparent operon (Pfam00361) | (204, 205) |
| SulP (Pfam00916) | Member of C or D clades of SulP-family transporters (Fig. S1); 8-12 predicted transmembrane helices | (206-209) |
| SbtA (Pfam05982) | 5-10 predicted transmembrane helices | (210, 211) |
| **Carbonic anhydrases** | **Active site residues** |  |
| Alpha CA (Pfam00194) | H94, H96, H119 corresponding to hCA1 (PDB 1CA2) | (212) |
| Beta CA (Pfam00484) | C403, H459, C462 corresponding to *P. purpureum* CA(PDB 1DDZ) | (212) |
| Gamma CA (Pfam00132) | H81, H117, H122 corresponding to *M. thermophila* CA (PDB 1THJ) | (212) |
| Delta CA (Pfam10563) | X-ABS indicates 3 H in active site. Active site residue location on *T. weissflogii* dCA is not known. All proteins included in this study had H40, H42, H47, H114, H254 residues corresponding to GenBank AAQ56178.1 | (213) |
| Zeta CA (Pfam18484) | C41, H93, C103 corresponding to *T. weissflogii* CA (PDB 3BOC) | (212) |
| Theta CA (Pfam18599) | C61, H117, C141 corresponding to *P. tricornutum* CA (PDB 5B5Y) | (212) |
| Iota CA (Pfam08332) | HHSS motif near carboxy termini | (214) |
| CsoSCA (Pfam08936) | C173, H242, C253 corresponding to *H. neapolitanus* CA (PDB 2FGY) | (212, 215) |

**
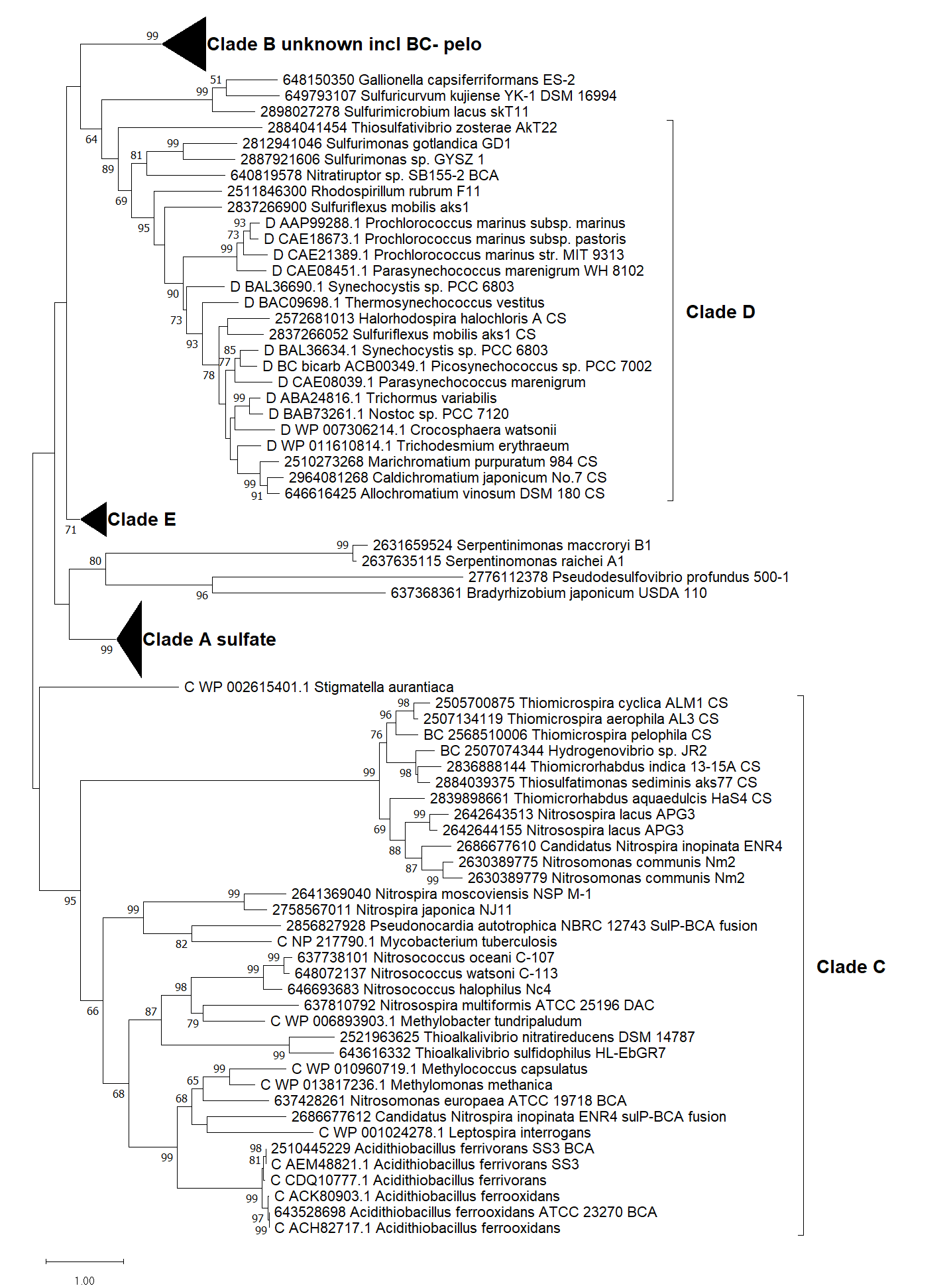
**

**FIG. S1.** Maximum likelihood analysis of members of the SulP family of transporters (Pfam00916) collected from genomes of autotrophic organisms studied here, and supplemented with sequences from genes encoding biochemically characterized transporters (BC), as well as those used by previous studies to identify five clades (A – E) of transporters (206-209). Clade A includes biochemically characterized sulfate transporters. Members of Clade B have not been biochemically characterized, and therefore have unknown substrate specificities. Clade C members are likely to all transport HCO_3_^-^; one member has been shown to transport HCO_3_^-^ (216), others are upregulated by low concentrations of dissolved inorganic carbon (209), and several are adjacent to carboxysome loci (CS), or genes encoding DAC transporters. Some are either adjacent to, or fused with, genes encoding carbonic anhydrase (217). Clade D includes HCO_3_^-^ transporters from cyanobacteria (208). Clade E includes a sequence originally suggested to encode a HCO_3_^-^ transporter, but subsequent study clarified that it transports succinate and other organic acids (218). The maximum likelihood method was implemented in MEGA 11 (202), using the Le Gascuel model (219) with a Gamma distribution and invariable sites. Taxon names include GenBank accession numbers or IMG gene object identifiers. Bootstrap values are based on 100 resamplings of the alignment.

**Calculating the amounts of CO_2_ and HCO_3_^-^ necessary to synthesize protein or nucleic acids for 1 gram dry weight (gdw) of biomass for a generic cell using different DIC fixation pathways**

A generic cell was used to illustrate the impact of different autotrophic DIC fixation pathways on the demand for CO_2_ and HCO_3_^-^ to synthesize biomass. An organism was selected from each of the 14 phyla represented in the set of genomes used for this study. Genome data from each of these 14 organisms was used to estimate the relative amounts of amino acids, ribonucleotides, and deoxyribonucleotides in their biomass, and these 14 values were in turn averaged to generate the estimates for the generic cell.

To begin the calculations for proteins, the amino acid sequences from all of the protein encoding genes from all 14 organisms were used to compute the average amino acid composition of proteins for each of the 14 species. These 14 amino acid compositions were averaged to generate an overall average amino acid composition for cell protein in the generic cell, which was used to estimate the amount of each amino acid necessary for 1 gdw, assuming proteins are 55% of dry weight (220).

To begin the calculations for deoxyribonucleotides, the GC content of genomic DNA from the 14 species selected above was averaged and used to estimate the quantity of deoxynucleotides (dAMP, dCMP, dGMP, and dTMP) necessary for 1 gram dry weight of generic cell biomass, assuming 3.1% of dry weight is DNA (220). For ribonucleotides for rRNA synthesis, the rRNA gene length and GC content were gathered from the genome sequences and were used to estimate the quantities of ribonucleotides (AMP, CMP, GMP, and UMP) needed to synthesize 1 gdw of generic cell biomass, assuming 17% of dry weight is rRNA (220). For ribonucleotides for tRNA synthesis, tRNA genes were gathered from the 14 genomes, and the average GC content of tRNA genes for each species was calculated. These 14 averages were in turn used to generate an average value for the generic cell, which was used to estimate the quantities of ribonucleotides needed to synthesize sufficient tRNA for 1 gdw of generic cell biomass, assuming 3% of dry weight is tRNA (220). For ribonucleotides for mRNA synthesis, the GC content of mRNA was assumed to be equal to the GC content of genomic DNA. The average genomic DNA GC content for a generic cell (calculated above) was used to estimate the quantities of ribonucleotides needed to synthesize sufficient mRNA for 1 gdw of generic cell biomass, assuming 0.8% of dry weight is mRNA (220). The ribonucleotides needed to synthesize rRNA, tRNA, and mRNA were combined to get an overall amount for total RNA.

From the quantity of amino acids and nucleotides, standard biosynthetic pathways were used to calculate the quantities of intermediates from central carbon metabolism (e.g., pyruvate, oxaloacetate) needed to synthesize the amino acids and nucleotides for proteins and nucleic acids for 1 gdw of generic cell biomass. Pathways from CO_2_ and HCO_3_^-^ (Fig. 6A) to each intermediate were used to calculate the relative amounts of both forms of DIC needed to synthesize the intermediate, and these values in turn were combined with the quantities of intermediates needed to synthesize proteins and nucleic acids calculated above to estimate the amounts of CO_2_ and HCO_3_^-^ to synthesize these macromolecules for 1 gdw generic cell biomass.

For the organisms in this study using the CBB, rTCA, WL, or DCHB, oxaloacetate is synthesized via carboxylation of phosphoenolpyruvate or pyruvate (Fig. 6). A number of enzymes can catalyze these carboxylations, and genes encoding all are present in the genomes of these organisms (Table S3).

**Table S3. Presence of genes encoding carboxylases capable of synthesizing oxaloacetate in the genomes of the autotrophic organisms studied here**

| **Enzyme** | **Number of genomes**  **encoding enzyme^a^ (213 genomes total)** |
| --- | --- |
| Phosphoenolpyruvate carboxylase (EC 4.1.1.31) | 108 |
| Pyruvate carboxylase (EC 6.4.1.1) | 124 |
| Oxaloacetate decarboxylase (Na^+^ extruding; EC 7.2.4.2) | 19 |
| Malic enzyme (EC 1.1.1.38, 1.1.1.39, 1.1.1.40)^b^ | 154 |
| Phosphoenolpyruvate carboxykinase  (EC 4.1.1.32, 4.1.1.38, 4.1.1.49) | 103 |

^a^Genes were found via EC number using KEGG Enzyme in IMG

^b^Malic enzyme could catalyze the carboxylation of pyruvate to form malate, which could then be oxidized to oxaloacetate via malate dehydrogenase

Under physiological conditions, pyruvate carboxylase and phosphoenolpyruvate carboxylase operate in the carboxylating direction (221, 222) , while phosphoenolpyruvate carboxykinase and malic enzyme generally operate in the decarboxylating direction (223), though it is important to note that there are exceptions where PEPCK has been shown to function as a carboxylase (224). Based on these observations, and the presence of phosphoenolpyruvate carboxylase and pyruvate carboxylase genes in 185 of the 188 organisms using the CBB, rTCA, WL, or DCHB pathways for carbon fixation, it was assumed for these calculations that oxaloacetate was synthesized by pyruvate carboxylase or phosphoenolpyruvate carboxylase, which use HCO_3_^-^ as a substrate (Table 1).

For most of the organisms using the rTCA, 2-oxoglutarate carboxylation is catalyzed by isocitrate dehydrogenase (which uses CO_2_); a few can also carboxylate 2-oxoglutarate via a biotin carboxylase (which uses HCO_3_^-^; (225). Since the majority use isocitrate dehydrogenase, the requirements of rTCA organisms for CO_2_ and HCO_3_^-^ were calculated assuming CO_2_ was used to carboxylate 2-oxoglutarate.

For the rTCA and DCHB, calculating the relative amounts of CO_2_ and HCO_3_^-^ necessary to synthesize biomass was complicated by an achiral intermediate (succinate) which scrambles CO_2_ and HCO_3_^-^ incorporation as C_1_ and C_4_ in this molecule. For both pathways, the calculations of the relative amounts of CO_2_ and HCO_3_^-^ in intermediates accommodated the scrambling at this step. Additionally, for the rTCA, the calculation is further complicated because the rTCA is two cycles simultaneously—an “acetyl-CoA rTCA” which begins with the carboxylation of acetyl-coA and produces oxaloacetate, and an “oxaloacetate rTCA” which begins with the reduction and eventual carboxylation of oxaloacetate, and produces acetyl-CoA. The origins of the carbon atoms of the organic acid intermediates of the rTCA from CO_2_ or HCO_3_^-^ depend on which cycle is used: repeated rounds of the oxaloacetate rTCA results in exclusive incorporation of CO_2_, since the two carboxylases within the oxaloacetate rTCA use CO_2_ as a substrate, while repeated rounds of the acetyl-CoA rTCA result in some HCO_3_^-^ incorporation due to the activity of phosphoenolpyruvate carboxylase or pyruvate carboxylase. The origin of carbon atoms in organic acids and other intermediates from the rTCA and gluconeogenesis was determined twice: once assuming exclusive acetyl-CoA rTCA operation, and once assuming exclusive oxaloacetate rTCA operation. To estimate the relative mix of intermediates from both cycles, the relative demand for intermediates derived from the acetyl-CoA rTCA and oxaloacetate rTCA were used to estimate the relative rates of the two cycles.

For pyrimidine biosynthesis, an additional HCO_3_^-^ per pyrimidine molecule was added to reflect the activity of carbamoyl phosphate synthase, EC 6.3.5.5 (226). For purine biosynthesis, the substrate used for carboxylation of aminoimidazole ribonucleotide (AIR) varies between organisms. Organisms with both *purK* and *purE* genes use HCO_3_^-^ to carboxylate AIR (227). Some genomes only encode PurE, which can carboxylate AIR using CO_2_ (228), suggesting that AIR carboxylation is via CO_2_ in these organisms (229). Most of the organisms in this study have both *purK* and *purE* genes, suggesting AIR carboxylation using HCO_3_^-^, and this has been used to calculate the relative amounts of CO_2_ and HCO_3_^-^ needed for nucleotide biosynthesis. However, most of the organisms using the WL pathway lack *purK* genes and may use CO_2_ for AIR carboxylation. Therefore, the amounts of CO_2_ and HCO_3_^-^ used for nucleotide biosynthesis for WL organisms was calculated using CO_2_ for AIR carboxylation, while this value for all of the other organisms was calculated using HCO_3_^-^ for AIR carboxylation.

**REFERENCES**

1. Bettstetter M, Peng X, Garrett RA, Prangishvili D. 2003. AFV1, a novel virus infecting hyperthermophilic archaea of the genus acidianus. Virology 315:68-79.

2. Yoshida N, Nakasato M, Ohmura N, Ando A, Saiki H, Ishii M, Igarashi Y. 2006. Acidianus manzaensis sp nov., a novel thermoacidophilic Archaeon growing autotrophically by the oxidation of H-2 with the reduction of Fe3+. Current Microbiology 53:406-411.

3. Khaleque HN, Gonzalez C, Kaksonen AH, Boxah NJ, Holmes DS, Watkin ELJ. 2019. Genome-based classification of two halotolerant extreme acidophiles, Acidihalobacter prosperus V6 (=DSM 14174=JCM 32253) and 'Acidihalobacter ferrooxidans' V8 (=DSM 14175=JCM 32254) as two new species, Acidihalobacter aeolianus sp. nov. and Acidihalobacter ferrooxydans sp. nov., respectively. International Journal of Systematic and Evolutionary Microbiology 69:1557-1565.

4. Pablo Cárdenas J, Ortiz R, Norris PR, Watkin E, Holmes DS. 2015. Reclassification of ‘Thiobacillus prosperus’ Huber and Stetter 1989 as Acidihalobacter prosperus gen. nov., sp. nov., a member of the family Ectothiorhodospiraceae. International Journal of Systematic and Evolutionary Microbiology 65:3641-3644.

5. Norris PR, Owen JP. 1993. Mineral sulphide oxidation by enrichment cultures of novel thermoacidophilic bacteria. FEMS Microbiology Reviews 11:51-56.

6. Kelly DP, Wood AP. 2000. Reclassification of some species of Thiobacillus to the newly designated genera Acidithiobacillus gen. nov., Halothiobacillus gen. nov. and Thermithiobacillus gen. nov. Int J Syst Evol Microbiol 50.

7. Hallberg KB, Gonzalez-Toril E, Johnson DB. 2010. Acidithiobacillus ferrivorans, sp. nov.; facultatively anaerobic, psychrotolerant iron-, and sulfur-oxidizing acidophiles isolated from metal mine-impacted environments. Extremophiles 14:9-19.

8. Meyer O, Schlegel HGn. 1978. Reisolation of the carbon monoxide utilizing hydrogen bacterium Pseudomonas carboxydovorans (Kistner) comb. nov. Archives of Microbiology 118:35-43.

9. Hoeft SE, Blum JS, Stolz JF, Tabita FR, Witte B, King GM, Santini JM, Oremland RS. 2007. Alkalilimnicola ehrlichii sp nov., a novel, arsenite-oxidizing haloalkaliphilic gammaproteobacterium capable of chemoautotrophic or heterotrophic growth with nitrate or oxygen as the electron acceptor. International Journal of Systematic and Evolutionary Microbiology 57:504-512.

10. Imhoff JF. 2005. Chromatiales ord. nov doi:10.1007/0-387-28022-7_1, p 1-59. Springer US.

11. Reysenbach A-L, Huber R, Stetter KO, Ishii M, Kawasumi T, Igarashi Y, Eder W, L’Haridon S, Jeanthon C. 2001. Phylum BI. Aquificae phy. nov doi:10.1007/978-0-387-21609-6_18, p 359-367. Springer New York.

12. Steinsbu BO, Thorseth IH, Nakagawa S, Inagaki F, Lever MA, Engelen B, Ovreas L, Pedersen RB. 2010. Archaeoglobus sulfaticallidus sp nov., a thermophilic and facultatively lithoautotrophic sulfate-reducer isolated from black rust exposed to hot ridge flank crustal fluids. International Journal of Systematic and Evolutionary Microbiology 60:2745-2752.

13. Ikuta T, Takaki Y, Nagai Y, Shimamura S, Tsuda M, Kawagucci S. 2016. Heterogeneous composition of key metabolic gene clusters in a vent mussel symbiont population. ISME J 10.

14. Ponnudurai R, Sayavedra L, Kleiner M, Heiden SE, Thurmer A, Felbeck H, Schluter R, Sievert SM, Daniel R, Schweder T, Markert S. 2017. Genome sequence of the sulfur-oxidizing Bathymodiolus thermophilus gill endosymbiont. Standards in Genomic Sciences 12.

15. Dubinina G, Savvichev A, Orlova M, Gavrish E, Verbarg S, Grabovich M. 2017. Beggiatoa leptomitoformis sp nov., the first freshwater member of the genus capable of chemolithoautotrophic growth. International Journal of Systematic and Evolutionary Microbiology 67:197-204.

16. Masuda S, Eda S, Ikeda S, Mitsui H, Minamisawa K. 2010. Thiosulfate-Dependent Chemolithoautotrophic Growth of <i>Bradyrhizobium japonicum</i>. Applied and Environmental Microbiology 76:2402-2409.

17. Delamuta JRM, Ribeiro RA, Ormeño-Orrillo E, Melo IS, Martínez-Romero E, Hungria M. 2013. Polyphasic evidence supporting the reclassification of Bradyrhizobium japonicum group Ia strains as Bradyrhizobium diazoefficiens sp. nov. International Journal of Systematic and Evolutionary Microbiology 63:3342-3351.

18. Saini MK, ChihChe W, Soulier N, Sebastian A, Albert I, Thiel V, Bryant DA, Hanada S, Tank M. 2020. Caldichromatium japonicum gen. nov., sp. nov., a novel thermophilic phototrophic purple sulphur bacterium of the Chromatiaceae isolated from Nakabusa hot springs, Japan. International Journal of Systematic and Evolutionary Microbiology 70:5701-5710.

19. Daebeler A, Herbold CW, Vierheilig J, Sedlacek CJ, Pjevac P, Albertsen M, Kirkegaard RH, de la Torre JR, Daims H, Wagner M. 2018. Cultivation and Genomic Analysis of "Candidatus Nitrosocaldus islandicus," an Obligately Thermophilic, Ammonia-Oxidizing Thaumarchaeon from a Hot Spring Biofilm in Graendalur Valley, Iceland. Frontiers in Microbiology 9.

20. Ahlgren NA, Chen YY, Needham DM, Parada AE, Sachdeva R, Trinh V, Chen T, Fuhrman JA. 2017. Genome and epigenome of a novel marine Thaumarchaeota strain suggest viral infection, phosphorothioation DNA modification and multiple restriction systems. Environmental Microbiology 19:2434-2452.

21. Santoro AE, Dupont CL, Richter RA, Craig MT, Carini P, McIlvin MR, Yang Y, Orsi WD, Moran DM, Saito MA. 2015. Genomic and proteomic characterization of "Candidatus Nitrosopelagicus brevis": An ammonia-oxidizing archaeon from the open ocean. Proceedings of the National Academy of Sciences of the United States of America 112:1173-1178.

22. Kim BK, Jung M-Y, Yu DS, Park S-J, Oh TK, Rhee S-K, Kim JF. 2011. Genome Sequence of an Ammonia-Oxidizing Soil Archaeon, “Candidatus Nitrosoarchaeum koreensis” MY1. Journal of Bacteriology 193:5539-5540.

23. Park SJ, Kim JG, Jung MY, Kim SJ, Cha IT, Ghai R, Martin-Cuadrado AB, Rodriguez-Valera F, Rhee SK. 2012. Draft Genome Sequence of an Ammonia-Oxidizing Archaeon, "Candidatus Nitrosopumilus sediminis" AR2, from Svalbard in the Arctic Circle. Journal of Bacteriology 194:6948-6949.

24. Zhalnina KV, Dias R, Leonard MT, de Quadros PD, Camargo FAO, Drew JC, Farmerie WG, Daroub SH, Triplett EW. 2014. Genome Sequence of Candidatus Nitrososphaera evergladensis from Group I.1b Enriched from Everglades Soil Reveals Novel Genomic Features of the Ammonia-Oxidizing Archaea. Plos One 9.

25. Hatzenpichler R, Lebedeva EV, Spieck E, Stoecker K, Richter A, Daims H, Wagner M. 2008. A moderately thermophilic ammonia-oxidizing crenarchaeote from a hot spring. Proceedings of the National Academy of Sciences of the United States of America 105:2134-2139.

26. Lehtovirta-Morley LE, Stoecker K, Vilcinskas A, Prosser JI, Nicol GW. 2011. Cultivation of an obligate acidophilic ammonia oxidizer from a nitrifying acid soil. Proceedings of the National Academy of Sciences 108:15892-15897.

27. Jung M-Y, Park S-J, Kim S-J, Kim J-G, Sinninghe Damsté JS, Jeon CO, Rhee S-K. 2014. A Mesophilic, Autotrophic, Ammonia-Oxidizing Archaeon of Thaumarchaeal Group I.1a Cultivated from a Deep Oligotrophic Soil Horizon. Applied and Environmental Microbiology 80:3645-3655.

28. Li Y, Ding K, Wen X, Zhang B, Shen B, Yang Y. 2016. A novel ammonia-oxidizing archaeon from wastewater treatment plant: Its enrichment, physiological and genomic characteristics. Scientific Reports 6.

29. Spieck E, Hartwig C, McCormack I, Maixner F, Wagner M, Lipski A, Daims H. 2006. Selective enrichment and molecular characterization of a previously uncultured Nitrospira-like bacterium from activated sludge. Environmental Microbiology 8:405-415.

30. Nowka B, Off S, Daims H, Spieck E. 2014. Improved isolation strategies allowed the phenotypic differentiation of two Nitrospira strains from widespread phylogenetic lineages. FEMS Microbiology Ecology 91.

31. Daims H, Lebedeva EV, Pjevac P, Han P, Herbold C, Albertsen M, Jehmlich N, Palatinszky M, Vierheilig J, Bulaev A, Kirkegaard RH, von Bergen M, Rattei T, Bendinger B, Nielsen PH, Wagner M. 2015. Complete nitrification by Nitrospira bacteria. Nature 528:504-+.

32. Russell SL, Pepper-Tunick E, Svedberg J, Byrne A, Castillo JR, Vollmers C, Beinart RA, Corbett-Detig R. 2020. Horizontal transmission and recombination maintain forever young bacterial symbiont genomes. Plos Genetics 16.

33. Newton IL, Woyke T, Auchtung TA, Dilly GF, Dutton RJ, Fisher MC, Fontanez KM, Lau E, Stewart FJ, Richardson PM, Barry KW, Saunders E, Detter JC, Wu D, Eisen JA, Cavanaugh CM. 2007. The *Calyptogena magnifica* chemoautotrophic symbiont genome. Science 315:998-1000.

34. Henkel JV, Vogts A, Werner J, Neu TR, Spröer C, Bunk B, Schulz-Vogt HN. 2021. Candidatus Sulfurimonas marisnigri sp. nov. and Candidatus Sulfurimonas baltica sp. nov., thiotrophic manganese oxide reducing chemolithoautotrophs of the class Campylobacteria isolated from the pelagic redoxclines of the Black Sea and the Baltic Sea. Systematic and Applied Microbiology 44:126155.

35. Eddie BJ, Wang Z, Malanoski AP, Hall RJ, Oh SD, Heiner C, Lin B, Strycharz-Glaven SM. 2016. ‘Candidatus Tenderia electrophaga', an uncultivated electroautotroph from a biocathode enrichment. International Journal of Systematic and Evolutionary Microbiology 66:2178-2185.

36. Peduzzi S, Storelli N, Welsh A, Peduzzi R, Hahn D, Perret X, Tonolla M. 2012. Candidatus "Thiodictyon syntrophicum", sp nov., a new purple sulfur bacterium isolated from the chemocline of Lake Cadagno forming aggregates and specific associations with Desulfocapsa sp. Systematic and Applied Microbiology 35:139-144.

37. Shah V, Morris RM. 2015. Genome Sequence of "Candidatus Thioglobus autotrophica" Strain EF1, a Chemoautotroph from the SUP05 Clade of Marine Gammaproteobacteria. Genome Announc 3.

38. Marshall KT, Morris RM. 2013. Isolation of an aerobic sulfur oxidizer from the SUP05/Arctic96BD-19 clade. The ISME Journal 7:452-455.

39. Kuwahara H, Yoshida T, Takaki Y, Shimamura S, Nishi S, Harada M, Matsuyama K, Takishita K, Kawato M, Uematsu K, Fujiwara Y, Sato T, Kato C, Kitagawa M, Kato I, Maruyama T. 2007. Reduced genome of the thioautotrophic intracellular symbiont in a deep-sea clam, *Calyptogena okutanii*. Curr Biol 17:881-6.

40. Imhoff JF. 2003. Phylogenetic taxonomy of the family Chlorobiaceae on the basis of 16S rRNA and fmo (Fenna Matthews-Olson protein) gene sequences. International Journal of Systematic and Evolutionary Microbiology 53:941-951.

41. Wahlund TM, Woese CR, Castenholz RW, Madigan MT. 1991. A thermophilic green sulfur bacterium from New Zealand hot springs, Chlorobium tepidum sp. nov. Archives of Microbiology 156:81-90.

42. Vogl K, Glaeser J, Pfannes KR, Wanner G, Overmann R. 2006. Chlorobium chlorochromatii sp nov., a symbiotic green sulfur bacterium isolated from the phototrophic consortium "Chlorochromatium aggregatum". Archives of Microbiology 185:363-372.

43. Pfennig N. 1968. Chlorobium phaeobacteroides nov. spec. und C. phaeovibrioides nov. spec., zwei neue Arten der grünen Schwefelbakterien. Archiv für Mikrobiologie 63:224-226.

44. Hanada S, Hiraishi A, Shimada K, Matsuura K. 1995. Chloroflexus aggregans sp. nov., a Filamentous Phototrophic Bacterium Which Forms Dense Cell Aggregates by Active Gliding Movement. International Journal of Systematic Bacteriology 45:676-681.

45. Pierson BK, Castenholz RW. 1974. PHOTOTROPIC GLIDING FILAMENTOUS BACTERIUM OF HOT SPRINGS, CHLOROFLEXUS-AURANTIACUS, GEN - AND SP-NOV. Archives of Microbiology 100:5-24.

46. Gibson J, Pfennig N, Waterbury JB. 1984. CHLOROHERPETON-THALASSIUM GEN-NOV ET SPEC NOV, A NON-FILAMENTOUS, FLEXING AND GLIDING GREEN SULFUR BACTERIUM. Archives of Microbiology 138:96-101.

47. Braun M, Mayer F, Gottschalk G. 1981. CLOSTRIDIUM-ACETICUM (WIERINGA), A MICROORGANISM PRODUCING ACETIC-ACID FROM MOLECULAR-HYDROGEN AND CARBON-DIOXIDE. Archives of Microbiology 128:288-293.

48. Makkar NS, Casida LE. 1987. Cupriavidus necator gen. nov., sp. nov.; a Nonobligate Bacterial Predator of Bacteria in Soil. International Journal of Systematic Bacteriology 37:323-326.

49. Finster K, Liesack W, Thamdrup B. 1998. Elemental Sulfur and Thiosulfate Disproportionation by <i>Desulfocapsa sulfoexigens</i> sp. nov., a New Anaerobic Bacterium Isolated from Marine Surface Sediment. Applied and Environmental Microbiology 64:119-125.

50. Visser M, Worm P, Muyzer G, Pereira IAC, Schaap PJ, Plugge CM, Kuever J, Parshina SN, Nazina TN, Ivanova AE, Bernier-Latmani R, Goodwin LA, Kyrpides NC, Woyke T, Chain P, Davenport KW, Spring S, Klenk H-P, Stams AJM. 2013. Genome analysis of Desulfotomaculum kuznetsovii strain 17T reveals a physiological similarity with Pelotomaculum thermopropionicum strain SIT. Standards in Genomic Sciences 8:69-87.

51. Brysch K, Schneider C, Fuchs G, Widdel F. 1987. Lithoautotrophic growth of sulfate-reducing bacteria, and description of Desulfobacterium autotrophicum gen. nov., sp. nov. Archives of Microbiology 148:264-274.

52. Schink B, Thiemann V, Laue H, Friedrich M. 2002. Desulfotignum phosphitoxidans sp. nov., a new marine sulfate reducer that oxidizes phosphite to phosphate. Archives of Microbiology 177:381-391.

53. L'Haridon S, Cilia V, Messner P, Raguenes G, Gambacorta A, Sleytr UB, Prieur D, Jeanthon C. 1998. Desulfurobacterium thermolithotrophum gen. nov., sp. nov., a novel autotrophic, sulphur-reducing bacterium isolated from a deep-sea hydrothermal vent. International Journal of Systematic Bacteriology 48:701-711.

54. Kato S, Krepski S, Chan C, Itoh T, Ohkuma M. 2014. Ferriphaselus amnicola gen. nov., sp nov., a neutrophilic, stalk-forming, iron-oxidizing bacterium isolated from an iron-rich groundwater seep. International Journal of Systematic and Evolutionary Microbiology 64:921-925.

55. Hafenbradl D, Keller M, Dirmeier R, Rachel R, Rossnagel P, Burggraf S, Huber H, Stetter KO. 1996. Ferroglobus placidus gen nov, sp nov, a novel hyperthermophilic archaeum that oxidizes Fe2+ at neutral pH under anoxic conditions. Archives of Microbiology 166:308-314.

56. Golyshina OV, Pivovarova TA, Karavaiko GI, Kondrat'eva TF, Moore ERB, Abraham WR, Lunsdorf H, Timmis KN, Yakimov MM, Golyshin PN. 2000. Ferroplasma acidiphilum gen. nov., sp nov., an acidophilic, autotrophic, ferrous-iron-oxidizing, cell-wall-lacking, mesophilic member of the Ferroplasmaceae fam. nov., comprising a distinct lineage of the Archaea. International Journal of Systematic and Evolutionary Microbiology 50:997-1006.

57. Emerson D, Moyer C. 1997. Isolation and characterization of novel iron-oxidizing bacteria that grow at circumneutral pH. Applied and Environmental Microbiology 63:4784-4792.

58. Slobodkina GB, Kolganova TV, Querellou J, Bonch-Osmolovskaya EA, Slobodkin AI. 2009. Geoglobus acetivorans sp. nov., an iron(III)-reducing archaeon from a deep-sea hydrothermal vent. INTERNATIONAL JOURNAL OF SYSTEMATIC AND EVOLUTIONARY MICROBIOLOGY 59:2880-2883.

59. Kashefi K, Tor JM, Holmes DE, Gaw Van Praagh CV, Reysenbach A-L, Lovley DR. 2002. Geoglobus ahangari gen. nov., sp. nov., a novel hyperthermophilic archaeon capable of oxidizing organic acids and growing autotrophically on hydrogen with Fe(III) serving as the sole electron acceptor. International Journal of Systematic and Evolutionary Microbiology 52:719-728.

60. Kelly DP, Wood AP. 2015. <i>Halothiobacillaceae fam. nov</i> doi:10.1002/9781118960608.fbm00221, p 1-2. Wiley.

61. Kawasumi T, Igarashi Y, Kodama T, Minoda Y. 1984. Hydrogenobacter thermophilus gen. nov., sp. nov., an Extremely Thermophilic, Aerobic, Hydrogen-Oxidizing Bacterium. International Journal of Systematic Bacteriology 34:5-10.

62. Romano C, D'Imperio S, Woyke T, Mavromatis K, Lasken R, Shock EL, McDermott TR. 2013. Comparative Genomic Analysis of Phylogenetically Closely Related Hydrogenobaculum sp Isolates from Yellowstone National Park. Applied and Environmental Microbiology 79:2932-2943.

63. Reysenbach A-L, Hamamura N, Podar M, Griffiths E, Ferreira S, Hochstein R, Heidelberg J, Johnson J, Mead D, Pohorille A, Sarmiento M, Schweighofer K, Seshadri R, Voytek MA. 2009. Complete and Draft Genome Sequences of Six Members of the <i>Aquificales</i>. Journal of Bacteriology 191:1992-1993.

64. Willems A, Busse J, Goor M, Pot B, Falsen E, Jantzen E, Hoste B, Gillis M, Kersters K, Auling G, Deley J. 1989. HYDROGENOPHAGA, A NEW GENUS OF HYDROGEN-OXIDIZING BACTERIA THAT INCLUDES HYDROGENOPHAGA-FLAVA COMB-NOV (FORMERLY PSEUDOMONAS-FLAVA), HYDROGENOPHAGA-PALLERONII (FORMERLY PSEUDOMONAS-PALLERONII), HYDROGENOPHAGA-PSEUDOFLAVA (FORMERLY PSEUDOMONAS-PSEUDOFLAVA AND PSEUDOMONAS-CARBOXYDOFLAVA), AND HYDROGENOPHAGA-TAENIOSPIRALIS (FORMERLY PSEUDOMONAS-TAENIOSPIRALIS)N. International Journal of Systematic Bacteriology 39:319-333.

65. Jannasch H, Wirsen C, Nelson D, Robertson L. 1985. Thiomicrospira crunogena sp. nov., a colorless, sulfur-oxidizing bacterium from a deep-sea hydrothermal vent. Int J Syst Bacteriol 35:422-424.

66. Boden R, Scott KM, Williams J, Russel S, Antonen K, Rae AW, Hutt LP. 2017. An evaluation of Thiomicrospira, Hydrogenovibrio and Thioalkalimicrobium: Reclassification of four species of Thiomicrospira to each Thiomicrorhabdus gen. nov. and Hydrogenovibrio, and reclassification of all four species of Thioalkalimicrobium to Thiomicrospira. Int J Syst Evol Micr 67:1140-1151.

67. Paper W, Jahn U, Hohn MJ, Kronner M, Näther DJ, Burghardt T, Rachel R, Stetter KO, Huber H. 2007. Ignicoccus hospitalis sp. nov., the host of ‘Nanoarchaeum equitans’. International Journal of Systematic and Evolutionary Microbiology 57:803-808.

68. Reiner JE, Jung T, Lapp CJ, Siedler M, Bunk B, Overmann J, Gescher J. 2018. Kyrpidia spormannii sp. nov., a thermophilic, hydrogen-oxidizing, facultative autotroph, isolated from hydrothermal systems at São Miguel Island, and emended description of the genus Kyrpidia. International Journal of Systematic and Evolutionary Microbiology 68:3735-3740.

69. Bonjour F, Aragno M. 1984. Bacillus tusciae, a new species of thermoacidophilic, facultatively chemolithoautotrophic hydrogen oxidizing sporeformer from a geothermal area. Archives of Microbiology 139:397-401.

70. Meyer JL, Huber JA. 2014. Strain-level genomic variation in natural populations of Lebetimonas from an erupting deep-sea volcano. Isme Journal 8:867-880.

71. Gao J, Zhang C-G, Wu X-L, Wang H-H, Qiu G-Z. 2007. Isolation and identification of a strain of Leptospirillum ferriphilum from an extreme acid mine drainage site. Annals of microbiology 57:171-176.

72. Hippe H. 2000. Leptospirillum gen. nov. (ex Markosyan 1972), nom. rev., including Leptospirillum ferrooxidans sp. nov. (ex Markosyan 1972), nom. rev. and Leptospirillum thermoferrooxidans sp. nov. (Golovacheva et al. 1992). International Journal of Systematic and Evolutionary Microbiology 50:501-503.

73. Bazylinski DA, Williams TJ, Lefèvre CT, Berg RJ, Zhang CL, Bowser SS, Dean AJ, Beveridge TJ. 2013. Magnetococcus marinus gen. nov., sp. nov., a marine, magnetotactic bacterium that represents a novel lineage (Magnetococcaceae fam. nov., Magnetococcales ord. nov.) at the base of the Alphaproteobacteria. Int J Syst Evol Microbiol 63:801-808.

74. Imhoff JF, Suling J, Petri R. 1998. Phylogenetic relationships among the Chromatiaceae, their taxonomic reclassification and description of the new genera Allochromatium, Halochromatium, Isochromatium, Marichromatium, Thiococcus, Thiohalocapsa and Thermochromatium. International Journal of Systematic Bacteriology 48:1129-1143.

75. Chiu BK, Kato S, McAllister SM, Field EK, Chan CS. 2017. Novel Pelagic Iron-Oxidizing Zetaproteobacteria from the Chesapeake Bay Oxic-Anoxic Transition Zone. Frontiers in Microbiology 8.

76. Liu LJ, You XY, Guo X, Liu SJ, Jiang CY. 2011. Metallosphaera cuprina sp nov., an acidothermophilic, metal-mobilizing archaeon. International Journal of Systematic and Evolutionary Microbiology 61:2395-2400.

77. Huber G, Spinnler C, Gambacorta A, Stetter KO. 1989. Metallosphaera sedula gen, and sp. nov. Represents a New Genus of Aerobic, Metal-Mobilizing, Thermoacidophilic Archaebacteria. Systematic and Applied Microbiology 12:38-47.

78. Balch WE, Fox GE, Magrum LJ, Woese CR, Wolfe RS. 1979. Methanogens: reevaluation of a unique biological group. Microbiological Reviews 43:260-296.

79. Cadillo-Quiroz H, Brauer SL, Goodson N, Yavitt JB, Zinder SH. 2014. Methanobacterium paludis sp nov and a novel strain of Methanobacterium lacus isolated from northern peatlands. International Journal of Systematic and Evolutionary Microbiology 64:1473-1480.

80. Zhao HX, Wood AG, Widdel F, Bryant MP. 1988. AN EXTREMELY THERMOPHILIC METHANOCOCCUS FROM A DEEP-SEA HYDROTHERMAL VENT AND ITS PLASMID. Archives of Microbiology 150:178-183.

81. Jeanthon C, L'Haridon S, Reysenbach AL, Vernet M, Messner P, Sleytr UB, Prieur D. 1998. Methanococcus infernos sp. nov., a novel hyperthermophilic lithotrophic methanogen isolated from a deep-sea hydrothermal vent. International Journal of Systematic Bacteriology 48:913-919.

82. Jones WJ, Leigh JA, Mayer F, Woese CR, Wolfe RS. 1983. Methanococcus jannaschii sp. nov., an extremely thermophilic methanogen from a submarine hydrothermal vent. Archives of Microbiology 136:254-261.

83. Jeanthon C, L'Haridon S, Reysenbach AL, Corre E, Vernet M, Messner P, Sleytr UB, Prieur D. 1999. Methanococcus vulcanius sp. nov., a novel hyperthermophilic methanogen isolated from East Pacific Rise, and identification of Methanococcus sp. DSM 4213(T) as Methanococcus fervens sp. nov. International Journal of Systematic Bacteriology 49:583-589.

84. Sakai S, Conrad R, Liesack W, Imachi H. 2010. Methanocella arvoryzae sp nov., a hydrogenotrophic methanogen isolated from rice field soil. International Journal of Systematic and Evolutionary Microbiology 60:2918-2923.

85. Kendall MM, Liu Y, Sieprawska-Lupa M, Stetter KO, Whitman WB, Boone DR. 2006. Methanococcus aeolicus sp nov., a mesophilic, methanogenic archaeon from shallow and deep marine sediments. International Journal of Systematic and Evolutionary Microbiology 56:1525-1529.

86. Jones WJ, Paynter MJB, Gupta R. 1983. CHARACTERIZATION OF METHANOCOCCUS-MARIPALUDIS SP-NOV, A NEW METHANOGEN ISOLATED FROM SALT-MARSH SEDIMENT. Archives of Microbiology 135:91-97.

87. Wilharm T, Zhilina TN, Hummel P. 1991. DNA-DNA HYBRIDIZATION OF METHYLOTROPHIC HALOPHILIC METHANOGENIC BACTERIA AND TRANSFER OF METHANOCOCCUS-HALOPHILUSVP TO THE GENUS METHANOHALOPHILUS AS METHANOHALOPHILUS-HALOPHILUS COMB-NOV. International Journal of Systematic Bacteriology 41:558-562.

88. Kurr M, Huber R, König H, Jannasch HW, Fricke H, Trincone A, Kristjansson JK, Stetter KO. 1991. Methanopyrus kandleri, gen. and sp. nov. represents a novel group of hyperthermophilic methanogens, growing at 110°C. Archives of Microbiology 156:239-247.

89. Bräuer SL, Cadillo-Quiroz H, Ward RJ, Yavitt JB, Zinder SH. 2011. Methanoregula boonei gen. nov., sp. nov., an acidiphilic methanogen isolated from an acidic peat bog. International Journal of Systematic and Evolutionary Microbiology 61:45-52.

90. Bryant MP, Boone DR. 1987. Emended Description of Strain MST(DSM 800T), the Type Strain of Methanosarcina barkeri. International Journal of Systematic Bacteriology 37:169-170.

91. Ferry JG, Smith PH, Wolfe RS. 1974. Methanospirillum, a New Genus of Methanogenic Bacteria, and Characterization of Methanospirillum hungatii sp.nov. International Journal of Systematic Bacteriology 24:465-469.

92. Wasserfallen A, Nölling J, Pfister P, Reeve J, Conway de Macario E. 2000. Phylogenetic analysis of 18 thermophilic Methanobacterium isolates supports the proposals to create a new genus, Methanothermobacter gen. nov., and to reclassify several isolates in three species, Methanothermobacter thermautotrophicus comb. nov., Methanothermobacter wolfeii comb. nov., and Methanothermobacter marburgensis sp. nov. International Journal of Systematic and Evolutionary Microbiology 50:43-53.

93. Sakai S, Takaki Y, Miyazaki M, Ogawara M, Yanagawa K, Miyazaki J, Takai K. 2019. Methanofervidicoccus abyssi gen. nov., sp. nov., a hydrogenotrophic methanogen, isolated from a hydrothermal vent chimney in the Mid-Cayman Spreading Center, the Caribbean Sea. International Journal of Systematic and Evolutionary Microbiology 69:1225-1230.

94. Takai K, Nealson KH, Horikoshi K. 2004. Methanotorris formicicus sp nov., a novel extremely thermophilic, methane-producing archaeon isolated from a black smoker chimney in the Central Indian Ridge. International Journal of Systematic and Evolutionary Microbiology 54:1095-1100.

95. Collins MD, Lawson PA, Willems A, Cordoba JJ, Fernandez-Garayzabal J, Garcia P, Cai J, Hippe H, Farrow JA. 1994. The phylogeny of the genus Clostridium: proposal of five new genera and eleven new species combinations. Int J Syst Bacteriol 44:812-26.

96. Smith JL, Campbell BJ, Hanson TE, Zhang CL, Cary SC. 2008. Nautilia profundicola sp. nov., a thermophilic sulfur-reducing epsilonproteobacterium from deep-sea hydrothermal vents. Int J Syst Evol Microbiol 58:1598-1602.

97. Smedile F, Foustoukos DI, Patwardhan S, Mullane K, Schlegel I, Adams MW, Schut GJ, Giovannelli D, Vetriani C. 2022. Adaptations to high pressure of Nautilia sp. strain PV-1, a piezophilic Campylobacterium (aka Epsilonproteobacterium) isolated from a deep-sea hydrothermal vent. Environmental Microbiology 24:6164-6183.

98. Nakagawa S, Takai K, Inagaki F, Horikoshi K, Sako Y. 2005. Nitratiruptor tergarcus gen. nov., sp. nov. and Nitratifractor salsuginis gen. nov., sp. nov., nitrate-reducing chemolithoautotrophs of the epsilonproteobacteria isolated from a deep-sea hydrothermal system in the Mid-Okinawa Trough. Int J Syst Evol Microbiol 55:925-933.

99. Shiotani T, Mino S, Sato W, Nishikawa S, Yonezawa M, Sievert SM, Sawabe T. 2020. Nitrosophilus alvini gen. nov., sp. nov., a hydrogen-oxidizing chemolithoautotroph isolated from a deep-sea hydrothermal vent in the East Pacific Rise, inferred by a genome-based taxonomy of the phylum “Campylobacterota”. PLOS ONE 15:e0241366.

100. Yoshida-Takashima Y, Takaki Y, Yoshida M, Zhang Y, Nunoura T, Takai K. 2022. Genomic insights into phage-host interaction in the deep-sea chemolithoautotrophic Campylobacterota, Nitratiruptor. ISME Communications 2:108.

101. Bock E, Sundermeyer-Klinger H, Stackebrandt E. 1983. New facultative lithoautotrophic nitrite-oxidizing bacteria. Archives of Microbiology 136:281-284.

102. Spieck E, Bock E. 2015. <i>Nitrobacter</i> doi:10.1002/9781118960608.gbm00803, p 1-11. Wiley.

103. Koops HP, Böttcher B, Möller UC, Pommerening-Röser A, Stehr G. 1990. Description of a new species of Nitrosococcus. Archives of Microbiology 154:244-248.

104. Watson SW. 1965. Characteristics of a marine nitrifying bacterium, Nitrosocystis oceanus sp. N. Limnol Oceanogr 10:R274-R289.

105. Campbell MA, Chain PS, Dang H, El Sheikh AF, Norton JM, Ward NL, Ward BB, Klotz MG. 2011. Nitrosococcus watsonii sp. nov., a new species of marine obligate ammonia-oxidizing bacteria that is not omnipresent in the world's oceans: calls to validate the names 'Nitrosococcus halophilus' and 'Nitrosomonas mobilis'. FEMS Microbiol Ecol 76:39-48.

106. Jung MY, Kim JG, Damste JSS, Rijpstra WIC, Madsen EL, Kim SJ, Hong H, Si OJ, Kerou M, Schleper C, Rhee SK. 2016. A hydrophobic ammonia-oxidizing archaeon of the Nitrosocosmicus clade isolated from coal tar-contaminated sediment. Environmental Microbiology Reports 8:983-992.

107. Koops HP, Bottcher B, Moller UC, Pommereningroser A, Stehr G. 1991. CLASSIFICATION OF 8 NEW SPECIES OF AMMONIA-OXIDIZING BACTERIA - NITROSOMONAS-COMMUNIS SP-NOV, NITROSOMONAS-UREAE SP-NOV, NITROSOMONAS-AESTUARII SP-NOV, NITROSOMONAS-MARINA SP-NOV, NITROSOMONAS-NITROSA SP-NOV, NITROSOMONAS-EUTROPHA SP-NOV, NITROSOMONAS-OLIGOTROPHA SP-NOV AND NITROSOMONAS-HALOPHILA SP-NOV. Journal of General Microbiology 137:1689-1699.

108. Nakagawa T, Takahashi R. 2015. Nitrosomonas stercoris sp nov., a Chemoautotrophic Ammonia-Oxidizing Bacterium Tolerant of High Ammonium Isolated from Composted Cattle Manure. Microbes and Environments 30:221-227.

109. Fukushi M, Mino S, Tanaka H, Nakagawa S, Takai K, Sawabe T. 2020. Biogeochemical Implications of N2O-Reducing Thermophilic Campylobacteria in Deep-Sea Vent Fields, and the Description of Nitratiruptor labii sp. nov. iScience 23:101462.

110. Bayer B, Vojvoda J, Reinthaler T, Reyes C, Pinto M, Herndl GJ. 2019. Nitrosopumilus adriaticus sp. nov. and Nitrosopumilus piranensis sp. nov., two ammonia-oxidizing archaea from the Adriatic Sea and members of the class Nitrososphaeria. International Journal of Systematic and Evolutionary Microbiology 69:1892-1902.

111. Qin W, Heal KR, Ramdasi R, Kobelt JN, Martens-Habbena W, Bertagnolli AD, Amin SA, Walker CB, Urakawa H, Konneke M, Devol AH, Moffett JW, Armbrust EV, Jensen GJ, Ingalls AE, Stahl DA. 2017. Nitrosopumilus maritimus gen. nov., sp nov., Nitrosopumilus cobalaminigenes sp nov., Nitrosopumilus oxyclinae sp nov., and Nitrosopumilus ureiphilus sp nov., four marine ammonia-oxidizing archaea of the phylum Thaumarchaeota. International Journal of Systematic and Evolutionary Microbiology 67:5067-5079.

112. Stieglmeier M, Klingl A, Alves RJE, Rittmann S, Melcher M, Leisch N, Schleper C. 2014. Nitrososphaera viennensis gen. nov., sp nov., an aerobic and mesophilic, ammonia-oxidizing archaeon from soil and a member of the archaeal phylum Thaumarchaeota. International Journal of Systematic and Evolutionary Microbiology 64:2738-2752.

113. Urakawa H, Garcia JC, Nielsen JL, Le VQ, Kozlowski JA, Stein LY, Lim CK, Pommerening-Roser A, Martens-Habbena W, Stahl DA, Klotz MG. 2015. Nitrosospira lacus sp nov., a psychrotolerant, ammonia-oxidizing bacterium from sandy lake sediment. International Journal of Systematic and Evolutionary Microbiology 65:242-250.

114. Watson SW, Graham LB, Remsen CC, Valois FW. 1971. LOBULAR, AMMONIA-OXIDIZING BACTERIUM, NITROSOLOBUS-MULTIFORMIS NOV-GEN NOV-SP. Archiv Fur Mikrobiologie 76:183-&.

115. Ushiki N, Fujitani H, Aoi Y, Tsuneda S. 2013. Isolation of Nitrospira belonging to Sublineage II from a Wastewater Treatment Plant. Microbes and Environments 28:346-353.

116. Ehrich S, Behrens D, Lebedeva E, Ludwig W, Bock E. 1995. A new obligately chemolithoautotrophic, nitrite-oxidizing bacterium,Nitrospira moscoviensis sp. nov. and its phylogenetic relationship. Archives of Microbiology 164:16-23.

117. Fujitani H, Momiuchi K, Ishii K, Nomachi M, Kikuchi S, Ushiki N, Sekiguchi Y, Tsuneda S. 2020. Genomic and Physiological Characteristics of a Novel Nitrite-OxidizingNitrospiraStrain Isolated From a Drinking Water Treatment Plant. Frontiers in Microbiology 11.

118. Keppen OI, Tourova TP, Kuznetsov BB, Ivanovsky RN, Gorlenko VM. 2000. Proposal of Oscillochloridaceae fam. nov, on the basis of a phylogenetic analysis of the filamentous anoxygenic phototrophic bacteria, and emended description of Oscillochloris and Oscillochloris trichoides in comparison with further new isolates. International Journal of Systematic and Evolutionary Microbiology 50:1529-1537.

119. Pfennig N, Overmann J. Chlorobium, p 1-9, Bergey's Manual of Systematics of Archaea and Bacteria doi:<https://doi.org/10.1002/9781118960608.gbm00374>.

120. Götz D, Banta A, Beveridge TJ, Rushdi AI, Simoneit BRT, Reysenbach AL. 2002. Persephonella marina gen. nov., sp. nov. and Persephonella guaymasensis sp. nov., two novel, thermophilic, hydrogen-oxidizing microaerophiles from deep-sea hydrothermal vents. International Journal of Systematic and Evolutionary Microbiology 52:1349-1359.

121. Vila X, Guyoneaud R, Cristina XP, Figueras JB, Abella CA. 2002. Green sulfur bacteria from hypersaline Chiprana Lake (Monegros, Spain): habitat description and phylogenetic relationship of isolated strains. Photosynthesis Research 71:165-172.

122. Beatty JT, Overmann J, Lince MT, Manske AK, Lang AS, Blankenship RE, Van Dover CL, Martinson TA, Plumley FG. 2005. An obligately photosynthetic bacterial anaerobe from a deep-sea hydrothermal vent. Proceedings of the National Academy of Sciences 102:9306-9310.

123. Thiel V, Drautz-Moses DI, Purbojati RW, Schuster SC, Lindemann S, Bryant DA. 2017. Genome Sequence of Prosthecochloris sp. Strain HL-130-GSB from the Phylum Chlorobi. Genome Announcements 5.

124. Cao JW, Gayet N, Zeng X, Shao ZZ, Jebbar M, Alain K. 2016. Pseudodesulfovibrio indicus gen. nov., sp nov., a piezophilic sulfate-reducing bacterium from the Indian Ocean and reclassification of four species of the genus Desulfovibrio. International Journal of Systematic and Evolutionary Microbiology 66:3904-3911.

125. Takamiya A, Tubaki K. 1956. A NEW FORM OF STREPTOMYCES CAPABLE OF GROWING AUTOTROPHICALLY. Archiv Fur Mikrobiologie 25:58-64.

126. Mahendra S, Alvarez-Cohen L. 2005. Pseudonocardia dioxanivorans sp. nov., a novel actinomycete that grows on 1,4-dioxane. International Journal of Systematic and Evolutionary Microbiology 55:593-598.

127. Lassalle F, Dastgheib SMM, Zhao FJ, Zhang J, Verbarg S, Fruhling A, Brinkmann H, Osborne TH, Sikorski J, Balloux F, Didelot X, Santini JM, Petersen J. 2021. Phylogenomics reveals the basis of adaptation of Pseudorhizobium species to extreme environments and supports a taxonomic revision of the genus. Systematic and Applied Microbiology 44.

128. Völkl P, Huber R, Drobner E, Rachel R, Burggraf S, Trincone A, Stetter KO. 1993. Pyrobaculum aerophilum sp. nov., a novel nitrate-reducing hyperthermophilic archaeum. Appl Environ Microbiol 59:2918-26.

129. Huber R, Kristjansson JK, Stetter KO. 1987. Pyrobaculum gen. nov., a new genus of neutrophilic, rod-shaped archaebacteria from continental solfataras growing optimally at 100°C. Archives of Microbiology 149:95-101.

130. Slobodkina GB, Lebedinsky AV, Chernyh NA, Bonch-Osmolovskaya EA, Slobodkin AI. 2015. Pyrobaculum ferrireducens sp nov., a hyperthermophilic Fe(III)-, selenate- and arsenate-reducing crenarchaeon isolated from a hot spring. International Journal of Systematic and Evolutionary Microbiology 65:851-856.

131. Chan PP, Cozen AE, Lowe TM. 2013. Reclassification of Thermoproteus neutrophilus Stetter and Zillig 1989 as Pyrobaculum neutrophilum comb. nov based on phylogenetic analysis. International Journal of Systematic and Evolutionary Microbiology 63:751-754.

132. Hafenbradl D, Jannasch HW, Stetter KO, Blöchl E, Rachel R, Burggraf S. 1997. Pyrolobus fumarii , gen. and sp. nov., represents a novel group of archaea, extending the upper temperature limit for life to 113°C. Extremophiles 1:14-21.

133. Garrity GM, Bell JA, Lilburn T. 2015. <i>Rhodobacterales ord. nov</i> doi:10.1002/9781118960608.obm00072, p 1-1. Wiley.

134. Madigan MT, Jung DO, Woese CR, Achenbach LA. 2000. Rhodoferax antarcticus sp nov., a moderately psychrophilic purple nonsulfur bacterium isolated from an Antarctic microbial mat. Archives of Microbiology 173:269-277.

135. van Niel CB. 1944. THE CULTURE, GENERAL PHYSIOLOGY, MORPHOLOGY, AND CLASSIFICATION OF THE NON-SULFUR PURPLE AND BROWN BACTERIA. Bacteriological Reviews 8:1-118.

136. Favinger J, Stadtwald R, Gest H. 1989. Rhodospirillum centenum, sp. nov., a thermotolerant cyst-forming anoxygenic photosynthetic bacterium. Antonie van Leeuwenhoek 55:291-296.

137. Hansen TA, Veldkamp H. 1973. Rhodopseudomonas sulfidophila, nov. spec., a new species of the purple nonsulfur bacteria. Archiv für Mikrobiologie 92:45-58.

138. Willems A, Gillis M, De Ley J. 1991. Transfer of Rhodocyclus gelatinosus to Rubrivivax gelatinosus gen. nov., comb. nov., and Phylogenetic Relationships with Leptothrix, Sphaerotilus natans, Pseudomonas saccharophila, and Alcaligenes latus. International Journal of Systematic Bacteriology 41:65-73.

139. Flood BE, Jones DS, Bailey JV. 2015. Sedimenticola thiotaurini sp nov., a sulfur-oxidizing bacterium isolated from salt marsh sediments, and emended descriptions of the genus Sedimenticola and Sedimenticola selenatireducens. International Journal of Systematic and Evolutionary Microbiology 65:2522-2530.

140. Bird LJ, Kuenen JG, Osburn MR, Tomioka N, Ishii S, Barr C, Nealson KH, Suzuki S. 2021. Serpentinimonas gen. nov., Serpentinimonas raichei sp. nov., Serpentinimonas barnesii sp. nov. and Serpentinimonas maccroryi sp. nov., hyperalkaliphilic and facultative autotrophic bacteria isolated from terrestrial serpentinizing springs. International Journal of Systematic and Evolutionary Microbiology 71.

141. Emerson D, Rentz JA, Lilburn TG, Davis RE, Aldrich H, Chan C, Moyer CL. 2007. A novel lineage of proteobacteria involved in formation of marine Fe-oxidizing microbial mat communities. PLoS One 2:e667.

142. Pickering BS, Oresnik IJ. 2008. Formate-dependent autotrophic growth in Sinorhizobium meliloti. Journal of Bacteriology 190:6409-6418.

143. Kelly DP, McDonald IR, Wood AP. 2000. Proposal for the reclassification of Thiobacillus novellus as Starkeya novella gen. nov., comb. nov., in the alpha-subclass of the Proteobacteria. International Journal of Systematic and Evolutionary Microbiology 50:1797-1802.

144. Norris PR, Clark DA, Owen JP, Waterhouse S. 1996. Characteristics of Sulfobacillus acidophilus sp. nov. and other moderately thermophilic mineral-sulphide-oxidizing bacteria. Microbiology 142:775-783.

145. Garrity GM, Holt JG, Reysenbach A-L, Huber H, Stetter KO, Zillig W, Itoh T, Suzuki K-I, Sanchez PC, Nakase T, Huber R, Sako Y, Nomura N. 2001. Phylum Al. Crenarchaeota phy. nov doi:10.1007/978-0-387-21609-6_16, p 169-210. Springer New York.

146. Dai X, Wang HN, Zhang ZF, Li KA, Zhang XL, Mora-Lopez M, Jiang CY, Liu C, Wang L, Zhu YX, Hernandez-Ascencio W, Dong ZY, Huang L. 2016. Genome Sequencing of Sulfolobus sp A20 from Costa Rica and Comparative Analyses of the Putative Pathways of Carbon, Nitrogen, and Sulfur Metabolism in Various Sulfolobus Strains. Frontiers in Microbiology 7.

147. Kojima H, Watanabe T, Fukui M. 2016. Sulfuricaulis limicola gen. nov., sp. nov., a sulfur oxidizer isolated from a lake. International Journal of Systematic and Evolutionary Microbiology 66:266-270.

148. Kojima H, Shinohara A, Fukui M. 2015. Sulfurifustis variabilis gen. nov., sp nov., a sulfur oxidizer isolated from a lake, and proposal of Acidiferrobacteraceae fam. nov and Acidiferrobacterales ord. nov. International Journal of Systematic and Evolutionary Microbiology 65:3709-3713.

149. Kodama Y, Watanabe K. 2004. Sulfuricurvum kujiense gen. nov., sp. nov., a facultatively anaerobic, chemolithoautotrophic, sulfur-oxidizing bacterium isolated from an underground crude-oil storage cavity. International Journal of Systematic and Evolutionary Microbiology 54:2297-2300.

150. Kojima H, Mochizuki J, Fukui M. 2020. Sulfuriferula nivalis sp. nov., a sulfur oxidizer isolated from snow and emended description of Sulfuriferula plumbiphila. International Journal of Systematic and Evolutionary Microbiology 70:3273-3277.

151. Drobner E, Huber H, Rachel R, Stetter KO. 1992. Thiobacillus plumbophilus spec. nov., a novel galena and hydrogen oxidizer. Archives of Microbiology 157:213-217.

152. Kojima H, Fukui M. 2016. Sulfuriflexus mobilis gen. nov., sp nov., a sulfur-oxidizing bacterium isolated from a brackish lake sediment. International Journal of Systematic and Evolutionary Microbiology 66:3515-3518.

153. Aguiar P, Beveridge TJ, Reysenbach AL. 2004. Sulfurihydrogenibium azorense, sp nov., a thermophilic hydrogen-oxidizing microaerophile from terrestrial hot springs in the Azores. International Journal of Systematic and Evolutionary Microbiology 54:33-39.

154. Nakagawa S, Shtaih Z, Banta A, Beveridge TJ, Sako Y, Reysenbach AL. 2005. Sulfurihydrogenibium yellowstonense sp. nov., an extremely thermophilic, facultatively heterotrophic, sulfur-oxidizing bacterium from Yellowstone National Park, and emended descriptions of the genus Sulfurihydrogenibium, Sulfurihydrogenibium subterraneum and Sulfurihydrogenibium azorense. International Journal of Systematic and Evolutionary Microbiology 55:2263-2268.

155. Kojima H, Kanda M, Umezawa K, Fukui M. 2021. Sulfurimicrobium lacusgen. nov., sp. nov., a sulfur oxidizer isolated from lake water, and review of the familySulfuricellaceaeto show that it is not a later synonym ofGallionellaceae. Archives of Microbiology 203:317-323.

156. Kojima H, Kato Y, Watanabe T, Fukui M. 2022. Sulfurimonas aquatica sp. nov., a sulfur-oxidizing bacterium isolated from water of a brackish lake. Archives of Microbiology 204.

157. Inagaki F, Takai K, Hideki KI, Nealson KH, Horikishi K. 2003. Sulfurimonas autotrophica gen. nov., sp nov., a novel sulfur-oxidizing epsilon-proteobacterium isolated from hydrothermal sediments in the Mid-Okinawa Trough. International Journal of Systematic and Evolutionary Microbiology 53:1801-1805.

158. Takai K, Suzuki M, Nakagawa S, Miyazaki M, Suzuki Y, Inagaki F, Horikoshi K. 2006. *Sulfurimonas paralvinellae* sp nov., a novel mesophilic, hydrogen- and sulfur-oxidizing chemolithoautotroph within the Epsilonproteo-bacteria isolated from a deep-sea hydrothermal vent polychaete nest, reclassification of Thiomicrospira denitrificans as Sulfurimonas denitrificans comb. nov and emended description of the genus Sulfurimonas. Int J Syst Evol Microbiol 56:1725-1733.

159. Labrenz M, Grote J, Mammitzsch K, Boschker HTS, Laue M, Jost G, Glaubitz S, Jurgens K. 2013. Sulfurimonas gotlandica sp. nov., a chemoautotrophic and psychrotolerant epsilonproteobacterium isolated from a pelagic redoxcline, and an emended description of the genus Sulfurimonas. Int J Syst Evol Microbiol 63:4141-4148.

160. Wang S, Jiang L, Hu Q, Cui L, Zhu B, Fu X, Lai Q, Shao Z, Yang S. 2021. Characterization of Sulfurimonas hydrogeniphila sp. nov., a Novel Bacterium Predominant in Deep-Sea Hydrothermal Vents and Comparative Genomic Analyses of the Genus Sulfurimonas. Front Microbiol 12:626705.

161. Wang SS, Jiang LJ, Liu XW, Yang SP, Shao ZZ. 2020. Sulfurimonas xiamenensis sp. nov. and Sulfurimonas lithotrophica sp. nov., hydrogen- and sulfur-oxidizing chemolithoautotrophs within the Epsilonproteobacteria isolated from coastal sediments, and an emended description of the genus Sulfurimonas. International Journal of Systematic and Evolutionary Microbiology 70:2657-2663.

162. Wang Z, Wang S, Lai Q, Wei S, Jiang L, Shao Z. 2022. Sulfurimonas marina sp. nov., an obligately chemolithoautotrophic, sulphur-oxidizing bacterium isolated from a deep-sea sediment sample from the South China Sea. International Journal of Systematic and Evolutionary Microbiology 72.

163. Wang SS, Shao ZZ, Lai QL, Liu XW, Xie SB, Jiang LJ, Yang SP. 2021. Sulfurimonas sediminis sp. nov., a novel hydrogen- and sulfur-oxidizing chemolithoautotroph isolated from a hydrothermal vent at the Longqi system, southwestern Indian ocean. Antonie Van Leeuwenhoek International Journal of General and Molecular Microbiology 114:813-822.

164. Gevertz D, Telang AJ, Voordouw G, Jenneman GE. 2000. Isolation and Characterization of Strains CVO and FWKO B, Two Novel Nitrate-Reducing, Sulfide-Oxidizing Bacteria Isolated from Oil Field Brine. Appl Environ Microbiol 66:2491-2501.

165. Tsuboi K, Sakai HD, Nur N, Stedman KM, Kurosawa N, Suwanto A. 2018. Sulfurisphaera javensis sp nov., a hyperthermophilic and acidophilic archaeon isolated from Indonesian hot spring, and reclassification of Sulfolobus tokodaii Suzuki et al. 2002 as Sulfurisphaera tokodaii comb. nov. International Journal of Systematic and Evolutionary Microbiology 68:1907-1913.

166. Kojima H, Watanabe M, Fukui M. 2017. Sulfuritortus calidifontis gen. nov., sp. nov., a sulfur oxidizer isolated from a hot spring microbial mat. Int J Syst Evol Microbiol 67:1355-1358.

167. Kojima H, Watanabe M, Fukui M. 2017. Sulfurivermis fontis gen. nov., sp nov., a sulfur-oxidizing autotroph, and proposal of Thioprofundaceae fam. nov. International Journal of Systematic and Evolutionary Microbiology 67:3458-3461.

168. Inagaki F, Takai K, Nealson KH, Horikoshi K. 2004. Sulfurovum lithotrophicum gen. nov., sp nov., a novel sulfur-oxidizing chemolithoautotroph within the epsilon-Proteobacteria isolated from Okinawa Trough hydrothermal sediments. International Journal of Systematic and Evolutionary Microbiology 54:1477-1482.

169. Xie S, Wang S, Li D, Shao Z, Lai Q, Wang Y, Wei M, Han X, Jiang L. 2019. Sulfurovum indicum sp. nov., a novel hydrogen- and sulfur-oxidizing chemolithoautotroph isolated from a deep-sea hydrothermal plume in the Northwestern Indian Ocean. Int J Syst Evol Microbiol 71.

170. Nakagawa S, Takai K, Inagaki F, Hirayama H, Nunoura T, Horikoshi K, Sako Y. 2005. Distribution, phylogenetic diversity and physiological characteristics of epsilon-Proteobacteria in a deep-sea hydrothermal field. Environ Microbiol 7:1619-1632.

171. Eder W, Huber R. 2002. New isolates and physiological properties of the Aquificales and description of Thermocrinis albus sp nov. Extremophiles 6:309-318.

172. Huber R, Eder W, Heldwein S, Wanner G, Huber H, Rachel R, Stetter KO. 1998. Thermocrinis ruber gen. nov., sp. nov., A pink-filament-forming hyperthermophilic bacterium isolated from yellowstone national park. Appl Environ Microbiol 64:3576-83.

173. Moussard H, L'Haridon S, Tindall BJ, Banta A, Schumann P, Stackebrandt E, Reysenbach AL, Jeanthon C. 2004. Thermodesulfatator indicus gen. nov., sp. nov., a novel thermophilic chemolithoautotrophic sulfate-reducing bacterium isolated from the Central Indian Ridge. International Journal of Systematic and Evolutionary Microbiology 54:227-233.

174. Siebers B, Zaparty M, Raddatz G, Tjaden B, Albers S-V, Bell SD, Blombach F, Kletzin A, Kyrpides N, Lanz C, Plagens A, Rampp M, Rosinus A, von Jan M, Makarova KS, Klenk H-P, Schuster SC, Hensel R. 2011. The Complete Genome Sequence of Thermoproteus tenax: A Physiologically Versatile Member of the Crenarchaeota. PLoS ONE 6:e24222.

175. Vetriani C, Speck MD, Ellor SV, Lutz RA, Starovoytov V. 2004. Thermovibrio ammonificans sp. nov., a thermophilic, chemolithotrophic, nitrate-ammonifying bacterium from deep-sea hydrothermal vents. International Journal of Systematic and Evolutionary Microbiology 54:175-181.

176. Sorokin DY, Tourova T, apos, P y, Sjollema KA, Kuenen JG. 2003. Thialkalivibrio nitratireducens sp. nov., a nitrate-reducing member of an autotrophic denitrifying consortium from a soda lake. International Journal of Systematic and Evolutionary Microbiology 53:1779-1783.

177. Sorokin DY, Tourova TP, Lysenko AM, Mityushina LL, Kuenen JG. 2002. Thioalkalivibrio thiocyanoxidans sp. nov. and Thioalkalivibrio paradoxus sp. nov., novel alkaliphilic, obligately autotrophic, sulfur-oxidizing bacteria capable of growth on thiocyanate, from soda lakes. International Journal of Systematic and Evolutionary Microbiology 52:657-664.

178. Sorokin DY, Muntyan MS, Panteleeva AN, Muyzer G. 2012. Thioalkalivibrio sulfidiphilus sp. nov., a haloalkaliphilic, sulfur-oxidizing gammaproteobacterium from alkaline habitats. International Journal of Systematic and Evolutionary Microbiology 62:1884-1889.

179. Sorokin DY, Lysenko AM, Mityushina LL, Tourova TP, Jones BE, Rainey FA, Robertson LA, Kuenen GJ. 2001. *Thioalkalimicrobium aerophilum* gen. nov., sp. nov. and *Thioalkalimicrobium sibericum* sp. nov., and *Thioalkalivibrio versutus* gen. nov., sp. nov., *Thioalkalivibrio nitratis* sp.nov., novel and *Thioalkalivibrio denitrificans* sp. nov., novel obligately alkaliphilic and obligately chemolithoautotrophic sulfur-oxidizing bacteria from soda lakes. Int J Syst Evol Microbiol 51:565-80.

180. Kelly DP, Wood AP, Stackebrandt E. 2015. <i>Thiobacillus</i> doi:10.1002/9781118960608.gbm00969, p 1-10. Wiley.

181. Imhoff JF, Pfennig N. 2001. Thioflavicoccus mobilis gen. nov., sp nov., a novel purple sulfur bacterium with bacteriochlorophyll b. International Journal of Systematic and Evolutionary Microbiology 51:105-110.

182. Sorokin DY, Kovaleva OL, Tourova TP, Muyzer G. 2010. Thiohalobacter thiocyanaticus gen. nov., sp. nov., a moderately halophilic, sulfur-oxidizing gammaproteobacterium from hypersaline lakes, that utilizes thiocyanate. International Journal of Systematic and Evolutionary Microbiology 60:444-450.

183. Nunoura T, Takaki Y, Kazama H, Kakuta J, Shimamura S, Makita H, Hirai M, Miyazaki M, Takai K. 2014. Physiological and Genomic Features of a Novel Sulfur-Oxidizing Gammaproteobacterium Belonging to a Previously Uncultivated Symbiotic Lineage Isolated from a Hydrothermal Vent. PLOS ONE 9:e104959.

184. Kojima H, Fukui M. 2019. Thiomicrorhabdus aquaedulcis sp. nov., a sulfur-oxidizing bacterium isolated from lake water. Int J Syst Evol Microbiol 69:2849-2853.

185. Watanabe T, Kojima H, Umezawa K, Hori C, Takasuka TE, Kato Y, Fukui M. 2019. Genomes of Neutrophilic Sulfur-Oxidizing Chemolithoautotrophs Representing 9 Proteobacterial Species From 8 Genera. Frontiers in Microbiology 10.

186. Liu X, Jiang L, Hu Q, Lyu J, Shao Z. 2020. Thiomicrorhabdus indica sp. nov., an obligately chemolithoautotrophic, sulfur-oxidizing bacterium isolated from a deep-sea hydrothermal vent environment. Int J Syst Evol Microbiol 70:234-239.

187. Sorokin DY, Gorlenko VM, Tourova TP, Tsapin AI, Nealson KH, Kuenen GJ. 2002. *Thioalkalimicrobium cyclicum* sp. nov. and *Thioalkalivibrio jannaschii* sp. nov., novel species of haloalkaliphilic, obligately chemolithoautotrophic sulfur-oxidizing bacteria from hypersaline alkaline Mono Lake (California). Int J Syst Evol Microbiol 52:913-20.

188. Slyemi D, Moinier D, Brochier-Armanet C, Bonnefoy V, Johnson DB. 2011. Characteristics of a phylogenetically ambiguous, arsenic-oxidizing Thiomonas sp., Thiomonas arsenitoxydans strain 3As(T) sp nov. Archives of Microbiology 193:439-449.

189. London J. 1963. Thiobacillus intermedius nov.sp. Archiv für Mikrobiologie 46:329-337.

190. Maier S. 1984. Description of Thioploca ingrica sp. nov., nom. rev. International Journal of Systematic Bacteriology 34:344-345.

191. Høgslund S, Nielsen JL, Nielsen LP. 2010. Distribution, ecology and molecular identification of Thioploca from Danish brackish water sediments. FEMS Microbiology Ecology doi:10.1111/j.1574-6941.2010.00878.x:no-no.

192. Bryantseva I, Gorlenko VM, Kompantseva EI, Imhoff JF, Suling J, Mityushina L. 1999. Thiorhodospira sibirica gen. nov., sp. nov., a new alkaliphilic purple sulfur bacterium from a Siberian soda lake. International Journal of Systematic Bacteriology 49:697-703.

193. Mochizuki J, Kojima H, Fukui M. 2021. Thiosulfativibrio zosterae gen. nov. sp. nov., and Thiosulfatimonas sediminis gen. nov., sp. nov. Arch Microbiol 203:951-957.

194. Graber JR, Leadbetter JR, Breznak JA. 2004. Description of Treponema azotonutricium sp. nov. and Treponema primitia sp. nov., the first spirochetes isolated from termite guts. Appl Environ Microbiol 70:1315-20.

195. Baumgarten J, Reh M, Schlegel HG. 1974. Taxonomic studies on some gram-positive coryneform hydrogen bacteria. Archives of Microbiology 100:207-217.

196. Cleland WW, Andrews TJ, Gutteridge S, Hartman FC, Lorimer GH. 1998. Mechanism of Rubisco:  The Carbamate as General Base. Chemical Reviews 98:549-562.

197. Aoshima M, Ishii M, Igarashi Y. 2004. A novel enzyme, citryl-CoA synthetase, catalyzing the first step of the citrate cleavage reaction in Hydrogenobacter thermophilus TK-6. Molec Microbiol 52:751-761.

198. Sato Y, Hosokawa K, Fujimura R, Nishizawa T, Kamijo T, Ohta H. 2009. Nitrogenase activity (acetylene reduction) of an iron-oxidizing leptospirillum strain cultured as a pioneer microbe from a recent volcanic deposit on miyake-jima, Japan. Microbes Environ 24:291-6.

199. Berg I. 2011. Ecological aspects of the distribution of different autotrophic CO2 fixation pathways. Appl Environ Microb 77:1925-1936.

200. Berg I, Kockelkorn D, Ramos-Vera W, Say RF, Zarzycki J, Hugler M, Alber B, Fuchs G. 2010. Autotrophic carbon fixation in archaea. Nature Rev Microbiol 8:447-459.

201. Krogh A, Larsson B, von Heijne G, Sonnhammer EL. 2001. Predicting transmembrane protein topology with a hidden Markov model: application to complete genomes. J Mol Biol 305:567-80.

202. Tamura K, Stecher G, Kumar S. 2021. MEGA11: Molecular Evolutionary Genetics Analysis Version 11. Mol Biol Evol 38:3022-3027.

203. Edgar RC. 2004. MUSCLE: Multiple sequence alignment with high accuracy and high throughput. Nucl Acids Res 32:1792-1797.

204. Schmid S, Chaput D, Breitbart M, Hines R, Williams S, Gossett HK, Parsi SD, Peterson R, Whittaker RA, Tarver A, Scott KM. 2021. Dissolved Inorganic Carbon-Accumulating Complexes from Autotrophic Bacteria from Extreme Environments. J Bacteriol 203:e0037721.

205. Mangiapia M, MicrobialPhysiology USF, Brown T-RW, Chaput D, Haller E, Harmer TL, Hashemy Z, Keeley R, Leonard J, Mancera P, Nicholson D, Stevens S, Wanjugi P, Zabinski T, Pan C, Scott KM. 2017. Proteomic and mutant analysis of the CO2 concentrating mechanism of hydrothermal vent chemolithoautotroph Thiomicrospira crunogena. J Bacteriol 199:e00871-16.

206. Wang C, Sun B, Zhang X, Huang X, Zhang M, Guo H, Chen X, Huang F, Chen T, Mi H, Yu F, Liu L-N, Zhang P. 2019. Structural mechanism of the active bicarbonate transporter from cyanobacteria. Nature Plants 5:1184-1193.

207. Shelden MC, Howitt SM, Price GD. 2010. Membrane topology of the cyanobacterial bicarbonate transporter, BicA, a member of the SulP (SLC26A) family. Mol Membr Biol 27:12-22.

208. Price GD, Howitt SM. 2011. The cyanobacterial bicarbonate transporter BicA: its physiological role and the implications of structural similarities with human SLC26 transporters. Biochem Cell Biol 89:178-88.

209. Esparza M, Jedlicki E, Gonzalez C, Dopson M, Holmes DS. 2019. Effect of CO_2_ Concentration on Uptake and Assimilation of Inorganic Carbon in the Extreme Acidophile *Acidithiobacillus ferrooxidans*. Frontiers in Microbiology 10:15.

210. Liu X, Chen B, Lai Q, Shao Z, Jiang L. 2021. Thiomicrorhabdus sediminis sp. nov. and Thiomicrorhabdus xiamenensis sp. nov., novel sulfur-oxidizing bacteria isolated from coastal sediments and an emended description of the genus Thiomicrorhabdus. International Journal of Systematic and Evolutionary Microbiology 71.

211. Price GD, Shelden MC, Howitt SM. 2011. Membrane topology of the cyanobacterial bicarbonate transporter, SbtA, and identification of potential regulatory loops. Mol Membr Biol 28:265-75.

212. Matsuda Y, Nawaly H, Yoneda K. 2022. Carbonic Anhydrase, p 167-195, Blue Planet, Red and Green Photosynthesis doi:<https://doi.org/10.1002/9781119986782.ch6>.

213. Cox EH, McLendon GL, Morel FM, Lane TW, Prince RC, Pickering IJ, George GN. 2000. The active site structure of Thalassiosira weissflogii carbonic anhydrase 1. Biochemistry 39:12128-30.

214. Del Prete S, Nocentini A, Supuran CT, Capasso C. 2020. Bacterial iota-carbonic anhydrase: a new active class of carbonic anhydrase identified in the genome of the Gram-negative bacterium Burkholderia territorii. Journal of Enzyme Inhibition and Medicinal Chemistry 35:1060-1068.

215. Sawaya MR, Cannon GC, Heinhorst S, Tanaka S, Williams EB, Yeates TO, Kerfeld CA. 2006. The Structure of beta-Carbonic Anhydrase from the Carboxysomal Shell Reveals a Distinct Subclass with One Active Site for the Price of Two. J Biol Chem 281:7546-7555.

216. Scott KM, Leonard JM, Boden R, Chaput CD, Dennison C, Haller E, Harmer TL, Anderson A, Arnold T, Budenstein S, Brown R, Brand J, Byers J, Calarco J, Campbell T, Carter E, Chase M, Cole M, Dwyer D, Grasham J, Hanni C, Hazle A, Johnson C, Johnson R, Kirby B, Lewis K, Neumann B, Nguyen T, Charari JN, Morakinyo O, Olsson B, Roundtree S, Skjerve E, Ubaldini A, Whittaker R. 2019. Diversity in CO2-Concentrating Mechanisms among Chemolithoautotrophs from the Genera Hydrogenovibrio, Thiomicrorhabdus, and Thiomicrospira, Ubiquitous in Sulfidic Habitats Worldwide. Applied and Environmental Microbiology 85.

217. Felce J, Saier MH. 2004. Carbonic anhydrase fused to anion transporters of the SulP family: Evidence for a novel type of bicarbonate transporter. J Mol Microbiol Biotechnol 8:169-176.

218. Karinou E, Compton ELR, Morel M, Javelle A. 2013. The Escherichia coli SLC26 homologue YchM (DauA) is a C4-dicarboxylic acid transporter. Molecular Microbiology 87:623-640.

219. Le SQ, Gascuel O. 2008. An improved general amino acid replacement matrix. Mol Biol Evol 25:1307-20.

220. Neidhardt FC, Ingraham JL, Schaechter M. 1990. Composition and organization of the bacterial cell, p 1-29. *In* Neidhardt FC, Ingraham JL, Schaechter M (ed), Physiology of the bacterial cell: A molecular approach. Sinauer Associates, Inc., Sunderland.

221. Kai Y, Matsumura H, Izui K. 2003. Phosphoenolpyruvate carboxylase: three-dimensional structure and molecular mechanisms. Archives of Biochemistry and Biophysics 414:170-179.

222. Attwood PV, Cleland WW. 1986. Decarboxylation of oxalacetate by pyruvate carboxylase. Biochemistry 25:8191-8196.

223. Sauer U, Eikmanns BJ. 2005. The PEP-pyruvate-oxaloacetate node as the switch point for carbon flux distribution in bacteria. FEMS Microbiol Rev 29:765-794.

224. Reiskind JB, Bowes G. 1991. THE ROLE OF PHOSPHOENOLPYRUVATE CARBOXYKINASE IN A MARINE MACROALGA WITH C4-LIKE PHOTOSYNTHETIC CHARACTERISTICS. Proceedings of the National Academy of Sciences of the United States of America 88:2883-2887.

225. Aoshima M, Igarashi Y. 2006. A novel oxalosuccinate-forming enzyme involved in the reductive carboxylation of 2-oxoglutarate in Hydrogenobacter thermophilus TK-6. Molecular Microbiology 62:748-759.

226. Jones ME, Spector L. 1960. PATHWAY OF CARBONATE IN THE BIOSYNTHESIS OF CARBAMYL PHOSPHATE. Journal of Biological Chemistry 235:2897-2901.

227. Zhang Y, Morar M, Ealick SE. 2008. Structural biology of the purine biosynthetic pathway. Cell Mol Life Sci 65:3699-724.

228. Firestine SM, Poon S-W, Mueller EJ, Stubbe J, Davisson VJ. 1994. Reactions Catalyzed by 5-Aminoimidazole Ribonucleotide Carboxylases from Escherichia coli and Gallus gallus: A Case for Divergent Catalytic Mechanisms? Biochemistry 33:11927-11934.

229. Brown AM, Hoopes SL, White RH, Sarisky CA. 2011. Purine biosynthesis in archaea: variations on a theme. Biology Direct 6:21.
